# Phylogenetic relationships within the speciose family Characidae (Teleostei: Ostariophysi: Characiformes) based on multilocus analysis and extensive ingroup sampling

**DOI:** 10.1186/1471-2148-11-275

**Published:** 2011-09-26

**Authors:** Claudio Oliveira, Gleisy S Avelino, Kelly T Abe, Tatiane C Mariguela, Ricardo C Benine, Guillermo Ortí, Richard P Vari, Ricardo M Corrêa e Castro

**Affiliations:** 1 Dept. Morfologia, Instituto de Biociências, Universidade Estadual Paulista, Botucatu, São Paulo, Brazil; 2 Dept. Biological Sciences, The George Washington University, USA; 3 Dept. Vertebrate Zoology, Smithsonian Institution, National Museum of Natural History, USA; 4 Laboratório de Ictiologia de Ribeirão Preto (LIRP), Dept. Biologia, FFCLRP, Universidade de São Paulo, Ribeirão Preto, São Paulo, Brazil

## Abstract

**Background:**

With nearly 1,100 species, the fish family Characidae represents more than half of the species of Characiformes, and is a key component of Neotropical freshwater ecosystems. The composition, phylogeny, and classification of Characidae is currently uncertain, despite significant efforts based on analysis of morphological and molecular data. No consensus about the monophyly of this group or its position within the order Characiformes has been reached, challenged by the fact that many key studies to date have non-overlapping taxonomic representation and focus only on subsets of this diversity.

**Results:**

In the present study we propose a new definition of the family Characidae and a hypothesis of relationships for the Characiformes based on phylogenetic analysis of DNA sequences of two mitochondrial and three nuclear genes (4,680 base pairs). The sequences were obtained from 211 samples representing 166 genera distributed among all 18 recognized families in the order Characiformes, all 14 recognized subfamilies in the Characidae, plus 56 of the genera so far considered *incertae sedis *in the Characidae. The phylogeny obtained is robust, with most lineages significantly supported by posterior probabilities in Bayesian analysis, and high bootstrap values from maximum likelihood and parsimony analyses.

**Conclusion:**

A monophyletic assemblage strongly supported in all our phylogenetic analysis is herein defined as the Characidae and includes the characiform species lacking a supraorbital bone and with a derived position of the emergence of the hyoid artery from the anterior ceratohyal. To recognize this and several other monophyletic groups within characiforms we propose changes in the limits of several families to facilitate future studies in the Characiformes and particularly the Characidae. This work presents a new phylogenetic framework for a speciose and morphologically diverse group of freshwater fishes of significant ecological and evolutionary importance across the Neotropics and portions of Africa.

## Background

One of the largest components of the freshwater fish fauna world-wide is the order Characiformes with nearly 2,000 species now recognized from myriad drainages of the New World and Africa [1]. Over 300 characiform species have been described in the last decade, primarily from the Neotropics and the pace of descriptions of new species gives no sign of abating. The characiform faunas on the two sides of the Atlantic Ocean demonstrate pronounced asymmetry in terms of numbers of both species and supraspecific taxa. The African components of the order include *circa *220 recognized species. These range south from the Nile River basin in the deserts of North Africa through much of the rest of the continent, with maximum diversity in the wetter areas such as the Congo River Basin, West Africa and Lower Guinea. On the other side of the Atlantic Ocean, over 1,700 species are now recognized extending from the southwestern portions of the United States south through Mexico and Central and South America to central Chile and Argentina. Major drainage basins in South America are all home to large and taxonomically overlapping assemblages of characiform species. Characiforms inhabit a range of ecosystems extending from the swiftly flowing rivers and streams of the Andean piedmont and cordilleras of the Neotropics through to the lentic backwaters of lowland flood plains in the Americas and Africa. Within these habitats, characiforms range from dozens of miniature and diminutive species (*sensu *Weitzman and Vari [2]) through to hundreds of midsized to giant species. Among the larger forms, many are economically and ecologically important, with some dominant in various drainages in terms of the total fish biomass. These and other characiform species play key roles for intra-ecosystem energy flux and material cycling in lowland river systems and as ecosystem engineers (e.g. Prochilodontidae - [3,4]).

African characiforms are now apportioned among four families, the Alestidae, Citharinidae, Distichodontidae and Hepsetidae, with the Alestidae and Distichodontidae accounting for 95% of the species among those families [1]. A single Neotropical genus (*Chalceus*) has been assigned to the otherwise African family Alestidae [5]. In contrast, the more speciose assemblage of New World characiforms is split into 14 families (Acestrorhynchidae, Anostomidae, Characidae, Chilodontidae, Crenuchidae, Ctenoluciidae, Curimatidae, Cynodontidae, Erythrinidae, Gasteropelecidae, Hemiodontidae, Lebiasinidae, Parodontidae, and Prochilodontidae) [6]; with the Serrasalmidae also recognized as a family by some authors (e.g. [7]).

**Figure 1:**
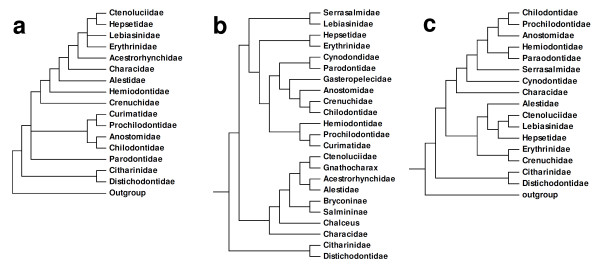
**Phylogenetic hypotheses for characiform families**. (a) morphological hypothesis modified from Buckup [8]; (b) molecular hypothesis (mitochondrial data) modified from Ortí and Meyer [30]; (c) molecular hypothesis (mitochondrial and nuclear data) modified from Calcagnotto *et al*. [32]. Note that these phylogenies differ in the number of families included.

To date, a single publication [8] has addressed higher level relationships across major components of the Characiformes based on morphological data. This study used 80 characters and 27 ingroup terminal taxa with representatives from all recognized characiform families except the Cynodontidae, Gasteropelecidae and Serrasalmidae (Figure 1a). Other morphologically based studies, although important, are more taxonomically restricted, focusing on phylogenetic questions ranging from the relationships among a few families through to relationships within families or their components. These included the Alestidae [5], Anostomidae [9,10], Characidae [11-23], Chilodontidae [9,24], Citharinidae [25], Crenuchidae [8], Curimatidae [9,26], Distichodontidae [25], Hemiodontidae [27] and Prochilodontidae [9,28].

Notwithstanding these efforts, no published hypothesis of phylogenetic relationships across characiforms based on an in-depth sampling of their morphological diversity is available. The most problematic group within this order is the family Characidae, whose composition and relationships within the Characiformes remains unsettled. With nearly 1,100 species, this family represents approximately 58% of the species within the Characiformes [1], and is the most active taxon in terms of new species descriptions (over 250 new species in the course of the last ten years). Considering the poor understanding of species-level diversity, particularly among miniature to small sized species, it is likely that the Characidae includes a disproportionate percentage of species yet to be described.

**Figure 2:**
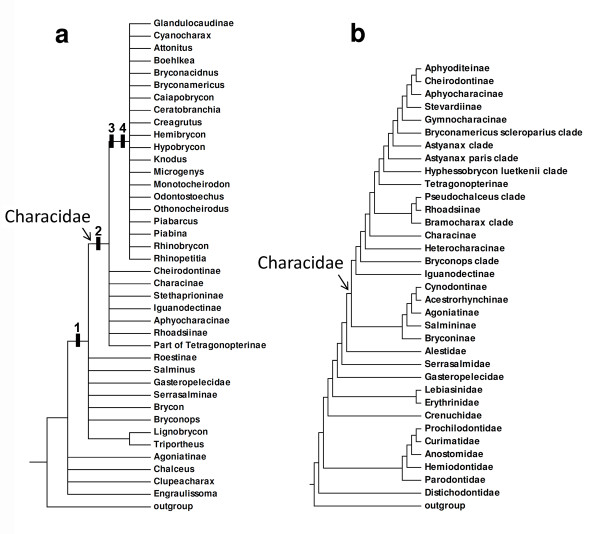
**Phylogenetic hypotheses among characids**. (a) morphological hypothesis modified from Malabarba and Weitzman [19]. Proposed synapomorphies: 1 - Presence of bony hooks on fin rays; 2 - Lack of supraorbital bone; 3 - Dorsal fin with two unbranched and eight branched rays (ii,8); 4 - Four teeth present on inner row of premaxilla. (b) morphological hypothesis modified from Mirande [23].

A classification of the family Characidae proposed by Reis *et al*. [6] highlighted some of these uncertainties. Twelve of the recognized subfamilies in that classification were assumed to represent monophyletic groups based on published results (Agoniatinae, Aphyocharacinae, Bryconinae, Characinae, Cheirodontinae, Clupeacharacinae, Glandulocaudinae, Iguanodectinae, Rhoadsiinae, Serrasalminae, Stethaprioninae and Tetragonopterinae). Nonetheless, 88 characid genera, many monotypic but others notably speciose (*Astyanax, Bryconamericus, Creagrutus, Hemigrammus, Hyphessobrycon, Jupiaba *and *Moenkhausia*), were placed as "*incertae sedis *in the Characidae" by Lima *et al*. [29]. Included among these *incertae sedis *were 620 of the 952 species assigned to the Characidae at that time. Concurrently, Malabarba and Weitzman [19] advanced a cladogram for the group based on four osteological features: (i) the presence of bony hooks on various fins, (ii) the absence of the supraorbital bone, (iii) the possession of two unbranched and eight branched rays in the dorsal fin, and (iv) the presence of four teeth in the inner tooth row of the premaxilla (Figure 2a). Of particular note is that the latter two characters delimited what Malabarba and Weitzman [19] identified as Clade A. This group included many of the genera considered to be *incertae sedis *in the Characidae by Lima *et al*. [29] along with taxa previously assigned to the Glandulocaudinae and Stevardiinae.

More recently, Mirande [22,23] advanced a comprehensive cladistic analysis for the Characidae based on 360 morphological characters scored for 160 characiform species. Although this study is the most comprehensive to date, it did not include representatives of 60 genera of the Characidae and or representatives from the characiform families Alestidae, Chilodontidae, Citharinidae, Ctenoluciidae and Hepsetidae. Interestingly, Mirande's [23] hypothesis (Figure 2b), obtained by weighted parsimony analysis, recovered the previously proposed Clade A of Malabarba and Weitzman [19] that he named Stevardiinae. On the other hand, Mirande's hypothesis [23] is incongruent with prior concepts of relationships among some taxa, with two examples of note being the close relationship among *Agoniates, Acestrorhynchus, Rhaphiodon *and *Salminus *and the inclusion of *Markiana *within *Astyanax*.

The first molecular investigations on this subject were published by Ortí and Meyer [30] and Ortí [31]. These were based on partial sequences of the mitochondrially-encoded 12S and 16S rRNA genes (about 880 bp) for 53 taxa. Although Ortí and Myer [30] noted that relationships among characiform families could not be reconstructed with confidence except for a few well-supported clades, their phylogenetic hypothesis (Figure 1b) suggested some interesting results. For example, they obtained a basal position of the African families Citharinidae and Distichodontidae, the hypothesis that the African and Neotropical characiform assemblages did not each constitute monophyletic groups and the finding that the Serrasalmidae is not closely related to groups then considered to form the family Characidae.

**Figure 3:**
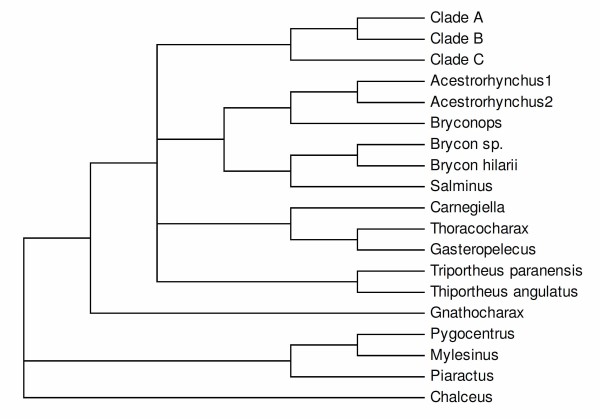
**Phylogenetic hypothesis for selected members of the Characidae proposed by Javonillo *et al***. [33]**based on molecular data**. Composition and relationships of clades A to C are discussed in the text.

Calcagnotto *et al*. [32] presented a more extensive molecular study of characiforms (Figure 1c) based on sequence analysis of two mitochondrial and four nuclear genes (about 3700 bp) for 124 characiform taxa (including 59 African representatives but excluding representatives of the Neotropical families Curimatidae and Gasteropelecidae). The large number of African taxa analyzed in that study supported the monophyletic nature of the families Citharinidae, Distichodontidae, Alestidae and Hepsetidae (the latter, however, being monotypic) and again rejected the concept of a monophyletic African assemblage. Five years later, Javonillo *et al*. [33] advanced a phylogenetic hypothesis for the Characidae (Figure 3) using DNA sequences of three mitochondrial genes and one nuclear gene. They analyzed 2940 bp for 98 taxa, including representatives of eight recognized subfamilies of the Characidae, and 33 genera considered *incertae sedis *in the Characidae by Lima *et al*. [29], plus the Acestrorhynchidae (2 species), Gasteropelecidae (3 species), and Serrasalmidae (3 genera). Their hypothesis supported three main clades within the Characidae (clades A, B, and C). Interestingly, all species included in clade A of Javonillo *et al*. [33] belong to Clade A of Malabarba & Weitzman [19], thus providing independent support for this hypothesis. Not surprisingly, however, representatives of some speciose and ill-defined genera such as *Astyanax, Bryconamericus, Hemigrammus*, and *Hyphessobrycon *appeared as polyphyletic.

Partial taxonomic overlap and use of disparate molecular markers in each of these studies has hindered combination of data sets for a more comprehensive analysis. Attempting to improve our knowledge and to test previous hypotheses of relationships among members of the Characidae, the present study is based on a broad taxon sampling (including all major lineages within the Characiformes and the Characidae) and a large molecular dataset with sequence data from two mitochondrial and three nuclear genes, with greater overlap with published data.

## Methods

### Taxon sampling

We follow the classification of the Characiformes with 18 families proposed by Nelson [1] as a framework for the selection of species included in this analysis: Acestrorhynchidae, Alestiidae (Alestidae), Anostomidae, Characidae (including the subfamilies Agoniatinae, Aphyocharacinae, Bryconinae, Characinae, Cheirodontinae, Clupeacharacinae, Glandulocaudinae, Iguanodectinae, Rhoadsiinae, Stethaprioninae, Tetragonopterinae), Chilodontidae, Citharinidae, Crenuchidae, Ctenoluciidae, Curimatidae, Cynodontidae, Distichodontidae, Erythrinidae, Gasteropelecidae, Hemiodontidae, Hepsetidae, Lebiasinidae, Parodontidae, and Prochilodontidae. We additionally recognize the family Serrasalmidae and in the Characidae, the subfamilies Stevardiinae as proposed by Weitzman *et al*. [20] and Triportheinae as proposed by Buckup [34] in selecting taxa for the present study. According to this classification, taxonomic sampling for the ingroup included 127 specimens representing all 13 recognized subfamilies of Characidae listed above, as well as 54 genera considered *incertae sedis *in the Characidae by Lima *et al*. [29], plus an undescribed characid genus. To study the delimitation of Characidae and its affinities with other families of Characiformes, we compiled a large and diverse outgroup (76 specimens) representing all 18 families of Characiformes, plus two genera of Cypriniformes to root the Characiform phylogeny. A complete list of taxa (205 specimens) is presented in Additional file 1. Tissue samples were primarily obtained from fish collected during the course of this study, supplemented by material obtained through the aquarium trade, or kindly donated by colleagues.

### DNA extraction and sequencing

Total DNA was extracted from ethanol preserved muscle, fin, and liver samples with the DNeasy Tissue Kit (Qiagen), following manufacturer's instructions. Partial sequences of the genes 16S rRNA, cytochrome *b *(Cytb), Myosin, heavy chain 6, cardiac muscle, alpha (Myh6), recombination activating gene 1 (RAG1), and recombination activating gene 2 (RAG2) were amplified by polymerase chain reaction (PCR) with the primers described in Additional file 2. Nested-PCRs were used to amplify the genes Myh6, Rag1, and Rag2 (Additional file 2). Amplifications were performed in a total volume of 25 μl with 2.5 μl of 10X buffer (10 mM Tris-HCl+15 mM MgCl_2_), 2.5 μl dNTPs (200 nM of each), 1 μl each 5 mM primer, 0.1 μl Taq Gold polymerase (Invitrogen), 1 μl template DNA (50 ng), and 17.9 μl ddH_2_O. The PCR reactions consisted of 35 cycles, 30 s at 95°C, 45-120 s at 48-58°C (according to primer and species), and 90 s at 72°C. All PCR products were first visually identified on a 1% agarose gel and then purified using ExoSap-IT^® ^(USB Corporation) following instructions of the manufacturer. The purified PCR products were sequenced using the "Big DyeTM Terminator v 3.1 Cycle Sequencing Ready Reaction Kit" (Applied Biosystems), purified again by ethanol precipitation and loaded on an automatic sequencer 3130-Genetic Analyzer (Applied Biosystems) in the Instituto de Biociências, Universidade Estadual Paulista, Botucatu, São Paulo. Contigs were assembled and edited in BioEdit 7.0.9.0 [35]. Where uncertainty of nucleotide identity was detected, IUPAC ambiguity codes were applied. Sequences have been deposited in GenBank (Additional file 3).

### Sequencing alignment and phylogenetic analyses

Sequences of each gene were independently aligned using the Muscle algorithm under default parameters [36] and the alignments inspected by eye for any obvious misalignments that were then corrected. A quality control step was included in our workflow to detect potential cases of sequencing errors due to contamination or paralogy. Alignments for each gene were initially analyzed by maximum likelihood (ML) [37] using the web servers RAxML BlackBox [38] to control for potential sequencing errors involving pseudogenes, paralogous copies or even laboratory cross-contamination or mistakes during the sequencing process. Sequences that were found misplaced in the resulting gene tree (as, for example, species of one family grouped with species of a obviously non-related family) were re-sequenced or eliminated from subsequent analyses. Given the degree of redundancy in taxonomic sampling, errors can be detected when sequences from putative congeneric or conspecific specimens are not placed together in the tree. This procedure was conducted only to check sequence quality and by the end of the study we found 11 CytB, 19 Myh6, 18 Rag1, and 24 Rag2 suspicious sequences that were excluded from the analysis. Genetic distances among sequences were calculated in Mega 5.04 [39]. To evaluate the occurrence of substitution saturation, we estimated the index of substitution saturation (Iss) in DAMBE 5.2.31 [40], as described by Xia *et al*. [41] and Xia and Lemey [42]. To investigate the relative contribution of each gene to the final phylogeny obtained by maximum likelihood analysis, we did a partitioned branch support analysis (PBS, [43,44]) using the program TreeRot [45] with 20 replicate heuristic searches.

Maximum parsimony (MP) analyses were conducted with PAUP* 4.0b10 [46]. Heuristic searches were performed with minimally 1000 random addition replicates and TBR branch swapping. All characters were unordered, all character transformations were equally weighted, and branches with maximum length of zero were collapsed. Gaps were treated as missing data. Clade robustness was assessed using 1000 bootstrap pseudoreplicates [47] with the same parameters as above.

RAxML [37] using the web servers RAxML BlackBox [38] was used for all maximum likelihood analyses using a mixed partition model. Random starting trees were used for each independent ML tree search and all other parameters were set on default values. All ML analyses were conducted under GTR +G since RAxML only applies this model [37]. Topological robustness was investigated using 1000 non-parametric bootstrap replicates.

Phylogenetic analyses using a partitioned Bayesian approach (BA) were conducted in MrBayes 3.1.2 [48]. A mixed model analysis was implemented, allowing individual models of nucleotide substitution to be estimated independently for each partition. A set of six reasonable partitioning schemes, ranging from 1 to 13 partitions (Table 1), was tested following the procedures outlined by Li *et al*. [49] under the AIC and BIC criteria. The best-fit model of nucleotide substitution was calculated in Paup* 4.0b10 [46] with the program Modeltest 3.7 [50] under default parameters using the Akaike information criterion [51, for justification]. Because MrBayes 3.1.2 only implements 1, 2, and 6 substitutions rate models, often it was not possible to implement the preferred model as selected by the AIC. In these situations, the nearest overparameterized model was used to avoid negative consequences of model violation or underparameterization [52,53]. As a consequence, the model for all partitions was set as: ''lset nst = 6" (GTR, TrN, TVM), ''rates = invgamma" (G + I), and the commands ''unlink" and ''prset ratepr = variable" were used to unlink model parameters across data partitions and define a rate multiplier for each partition. Two independent Bayesian analyses were conducted. Four independent MCMC chains were run with 30,000,000 replicates each, sampling one tree every 1000 steps. The distribution of log likelihood scores was examined to determine stationarity for each search and to decide if extra runs were required to achieve convergence, using the program Tracer 1.4 [54]. Initial trees estimated prior to convergence were discarded as part of a burn-in procedure, and the remaining trees were used to construct a 50% majority rule consensus tree in Paup* [46].

**Table 1:** Comparison of log likelihoods, AIC and BIC values among different partitioning schemes (from 1 to 13 partitions)

Number of partitions*	number of parameters	*L_ML_*	AIC	Δi	BIC_*ML*_
1	9	164,396	328,810	9894.730	328,825
2	19	162,890	325,819	6903.326	325,850
4A	39	163,108	326,295	7379.667	326,360
4B	39	161,931	323,941	5026.042	324,006
5	49	162,673	325,445	6529.681	325,527
13	129	159,328	318,915	0.000	319,131

For each type of analysis the following results are shown: total number of parameters; log likelihood calculated using RAxML (*L_ML_*); AIC values; the difference in AIC values among model i and the best model (Δ*i *= AIC*i *- AICmin); BIC_*ML *_values.

*1 partition = all dataset; 2 partitions = mitochondrial (16S + CytB) and nuclear (Myh6 + Rag1 + Rag2); 4 partitions A = 16S and 1st, 2nd, and 3rd codon position of protein coding genes; 4 partitions B = 16S + CytB and 1st, 2nd, and 3rd codon position of nuclear genes; 5 partitions = by each gene (16S + CytB + Myh6 + Rag1 + Rag2); 13 partitions = 16S + each codon position of each protein coding genes (1st, 2nd, and 3rd codon position of CytB; 1st, 2nd, and 3rd codon position of Myh6; 1st, 2nd, and 3rd codon position of Rag1; 1st, 2nd, and 3rd codon position of Rag2).

Alternative phylogenetic hypotheses were compared using likelihood-based tests implemented in the program Treefinder [55]. These tests assess the statistical significance of differences in likelihood scores between two or more hypotheses. Probabilities for alternative hypotheses were obtained for the Shimodaira-Hasegawa (SH) and the approximately unbiased (AU) tests [56,57]. Both testing procedures are adequate to compare hypotheses *a posteriori *based on the same data set, but since the SH test is more conservative [57], significance was determined when P-values obtained were P < 0.05 and P < 0.01 for SH and AU, respectively. Several hypotheses reflecting previous results (e.g., Lucena and Menezes [58], Calcagnotto *et al*. [32], Mirande [23]) and a set of alternative branching patters subtended by the basal nodes of the phylogeny obtained in this study were tested. Alternative hypotheses were constructed by performing tree-searches under specific topological constraints to find the ML tree that satisfies the branching pattern enforced. The constraints either fixed the topology or the composition for major clades, but in each case multifurcations within these clades or elsewhere in the tree were resolved by the tree search. Searches were conducted under ML using the program Treefinder with a 13-partition scheme and a GTR+G model independently optimized for each partition (the same approach used with RAxML). Results from each of these constrained tree searches were saved individually and subsequently joined into a single hypothesis file to perform the topology tests according to the Treefinder manual [55].

## Results

Partial sequences of two mitochondrial (16SrRNA and Cytb) and three nuclear genes (Myh6, Rag1 and Rag2) were obtained for 213 specimens (Additional file 3). The final matrix was deposited in TreeBase http://www.treebase.org under number 11474. Missing data, due to problems with PCR experiments, sequencing, or missing data in Genbank, corresponded to 11.7% of the total data set (Table 2). Data absence was more prevalent among nuclear (16.6% missing) than mitochondrial genes (5.0% missing), perhaps due to non-conserved priming regions and higher risk of cross-contamination in the nested PCR procedure. For each gene, the number and percentage of sequences obtained, their size (bp), number of variable sites, base pair composition, overall mean genetic distance (p-distance), the best substitution model for the gene, α (shape) parameter of Γ distribution, proportion of invariants (I) sites, number of informative characters under parsimony, and proportion of informative characters under parsimony are presented in Table 2. Under the MP criterion, about one-half of the positions were phylogenetically informative. The overall mean of genetic distance observed was between 0.087 ± 0.005 (Myh6) to 0.208 ± 0.007 (CytB), suggesting that the analyzed sequences have enough genetic variation for the phylogenetic studies of species, genera and families. Each gene and codon position partition was tested further to investigate the occurrence of substitution saturation [41,42], and the results showed that there is significant saturation only for the Rag2 3rd codon positions in the asymmetrical topology test (results not shown); however, considering that the Iss.c value is greater than the Iss value the information found in this position can be used in the phylogenetic analysis [41,42]. The best-fitting model of nucleotide substitution calculated for each partition was: GTR+ I+Γ (CytB 1st and 2nd position, Myh6 1st and 2nd position, Rag1 1st position), TVM+I+F (Myh6 3rd position, Rag1 2nd position, Rag2 1st position), K81uf+I+ Γ (Rag2 2nd position, Rag2 3rd position), TIM+I+ Γ (CytB 3rd position), TrN+I+ Γ (Rag1 3rd position).

**Table 2:** Information content and characteristics of each gene partition

			Gene		
	
	16S	CytB	Myh6	Rag1	Rag2
Number of sequences	213 (100%)	192 (90%)	178 (84%)	175 (82%)	182 (85%)
bp after alignment	633	992	755	1266	1034
Number of variable sites	357	636	377	835	680
Number of informative characters under parsimony	298	556	314	645	574
% informative characters under parsimony	47.07	56.04	41.59	50.94	55.51
*Π*_A_	0.2584	0.3472	0.3137	0.3059	0.2702
*Π*_C_	0.2186	0.3516	0.2205	0.1971	0.1961
*Π*_G_	0.1813	0.0623	0.1945	0.1947	0.2173
*Π*_T_	0.3418	0.2389	0.2713	0.3022	0.3164
Overall mean genetic distance (p-distance)	0.124 ± 0.007	0.208 ± 0.007	0.087 ± 0.005	0.111 ± 0.005	0.115 ± 0.004
Nucleotide substitution model	GTR	GTR	TrN	TVM	TVM
α (shape) parameter of Γ distribution	0.60	0.42	1.04	0.88	1.00
Proportion of invariants (I) sites	0.42	0.37	0.48	0.32	0.29

The combined data set contains significant phylogenetic information, given that most major lineages along the backbone of the tree were supported by high bootstrap values (> 70%). A partitioned Bremer support analysis was applied to the maximum likelihood majority rule consensus tree. The results show many positive scores, indicating positive contribution and some negative scores indicating conflicting signal for particular nodes. In general, the positive contributions were higher for the mitochondrial genes (especially 16S), than for nuclear genes (Table 3).

**Table 3:** Results of the partitioned Bremer support (PBS) analysis showing the percentage of nodes with positive values, indicating net positive contribution, observed for each gene in the final majority rule consensus ML tree

	16S	CytB	Myh6	Rag1	Rag2
All nodes	84.1	75.6	70.7	42.7	41.5
Familial nodes	85.7	71.4	81.0	57.1	42.9
Suprafamilial nodes	76.5	70.6	64.7	47.1	35.3
Infrafamilial nodes	86.4	79.5	68.2	34.1	43.2

Mean	83.2	74.3	71.2	45.3	40.7

Six different partitioning schemes, ranging from one to 13 partitions (Table 1), were tested to establish the optimal number of data partitions (following Li *et al*. [49]) for the final analysis. The results showed that the 13 partition model was the best choice (Table 1); however, ML analysis conducted with the other partitioning schemes resulted in the same final topology, with minor differences in branch length and support values (not shown).

**Figure 4:**
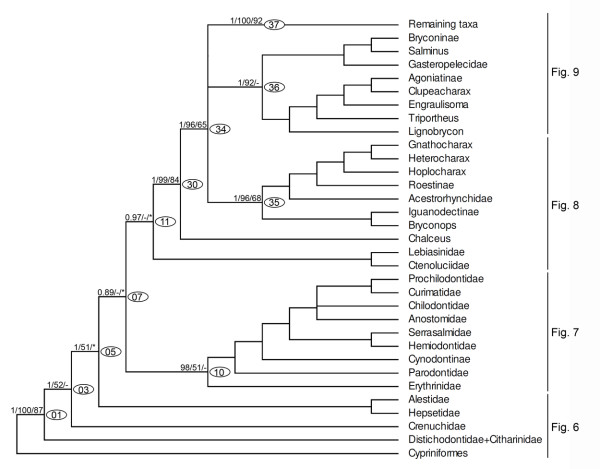
**Summary tree showing relationships among major lineages obtained by a maximum likelihood (ML) partitioned analysis of the concatenated dataset**. A series of three numbers (e.g., 1/100/87) at each of the main nodes represents the posterior probability for that split obtained in Bayesian analysis (B), percentage of bootstrap support obtained by ML, and percentage of bootstrap support obtained by MP analysis, respectively (1000 bootstrap replicates). Dashes represent values lower than 0.5 (B) or 50% (ML, MP). Nodes not supported by values higher than 0.5 (B) or 50% (ML, MP) were collapsed. Asterisks represent nodes that were not obtained by B or MP analyses. Clades labelled with numbers within ovals are discussed in the text and shown in subsequent figures.

**Figure 5:**
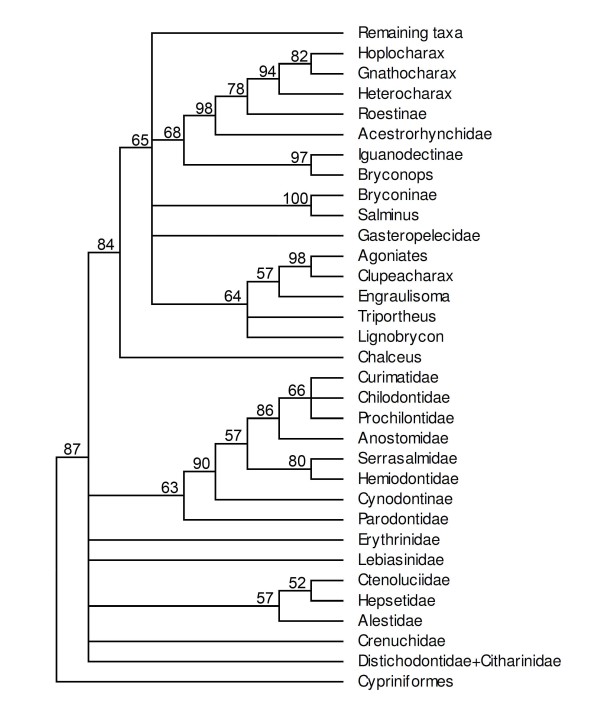
**Majority-rule consensus tree obtained in maximum parsimony analysis, showing an alternative hypothesis of relationships among some characiform families**. Numbers at nodes represent bootstrap supports.

**Figure 6:**
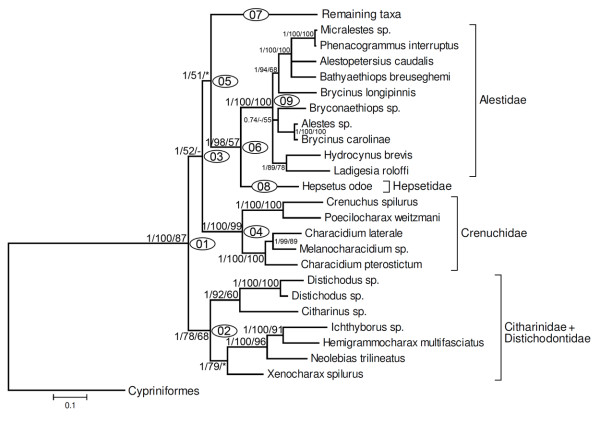
**Partial ML tree (see Figure 4 for a complete view) showing relationship among species of Citharinidae, Distichodontidae, Crenuchidae, Hepsetidae and Alestidae**. Numbered nodes as referenced in the text and values shown in Figure 4.

**Figure 7:**
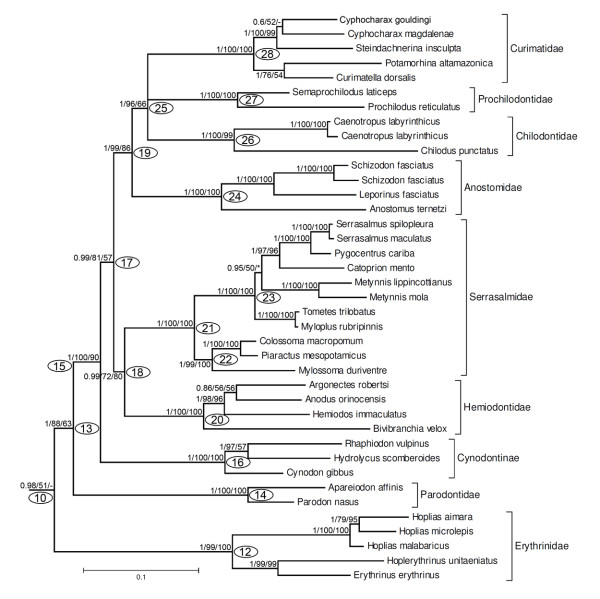
**Partial ML tree (see Figure 4 for a complete view) showing relationship among species of Erythrinidae, Parodontidae, Cynodontinae, Hemiodontidae, Serrasalmidae, Anostomidae, Chilodontidae, Prochilodontidae, and Curimatidae**. Numbered nodes as referenced in text and values as in Figure 4.

**Figure 8:**
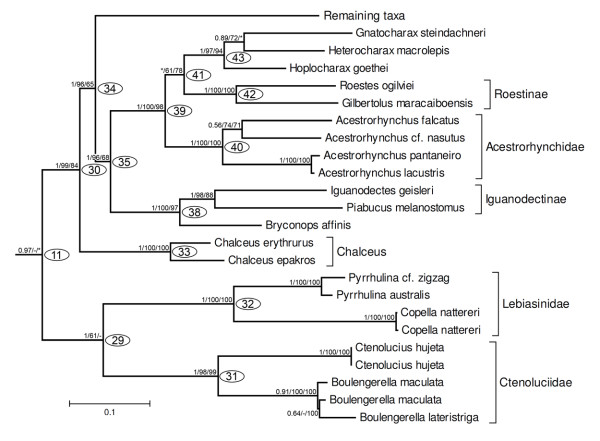
**Partial ML tree (see Figure 4 for a complete view) showing relationships among species of Ctenoluciidae, Lebiasinidae, *Chalceus, Bryconops*, Iguanodectinae, Acestrorhynchidae, Roestinae, *Heterocharax, Hoplocharax *and *Gnatocharax***. Numbered nodes as referenced in the text and values as Figure 4.

**Figure 9:**
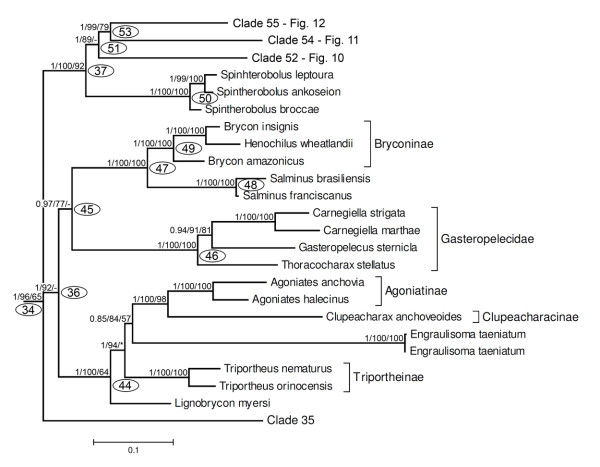
**Partial tree (see Figure 4 for a complete view) showing the relationship among species of Triportheinae, *Engraulisoma*, Clupeacharacinae, Agoniatinae, Gasteropelecidae, *Salminus*, Bryconinae, *Spintherobolus *and Clades 54, 56 and 57**. Numbered nodes as referenced in text with values in Figure 4.

**Figure 10:**
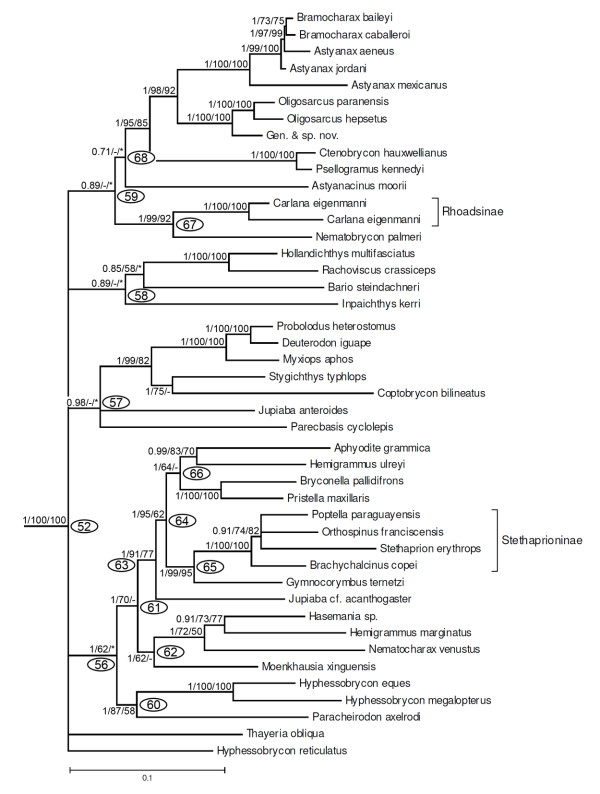
**Partial ML tree (see Figure 9 for overall view) showing relationships among species of the clade 54, including Stethaprioninae, Rhoadsiinae and several genera previously considered *incertae sedis *in Characidae**. Numbered nodes as referenced in text and values as in Figure 4.

**Figure 11:**
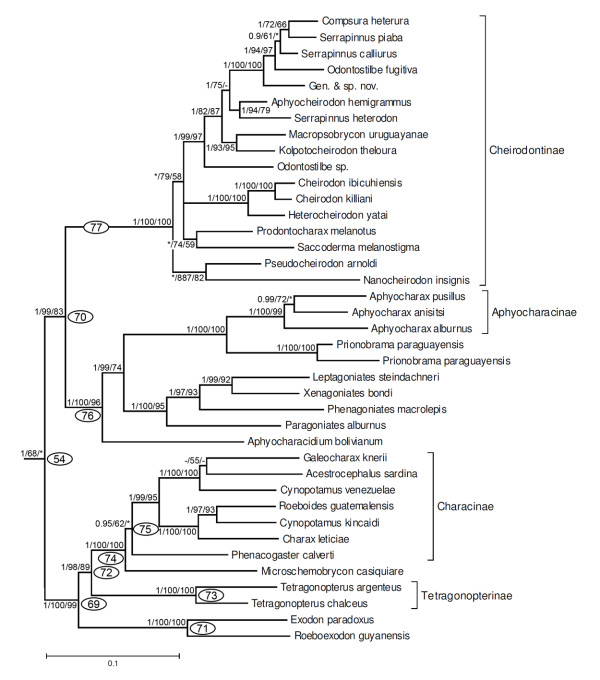
**Partial ML tree (see Figure 9 for overall view) showing relationships among species of the clade 56, including Cheirodontinae, Aphyocharacinae, Characinae, Tetragonopterinae and several genera previously considered *incertae sedis *in Characidae**. Numbered nodes as referenced in text and values as in Figure 4.

**Figure 12:**
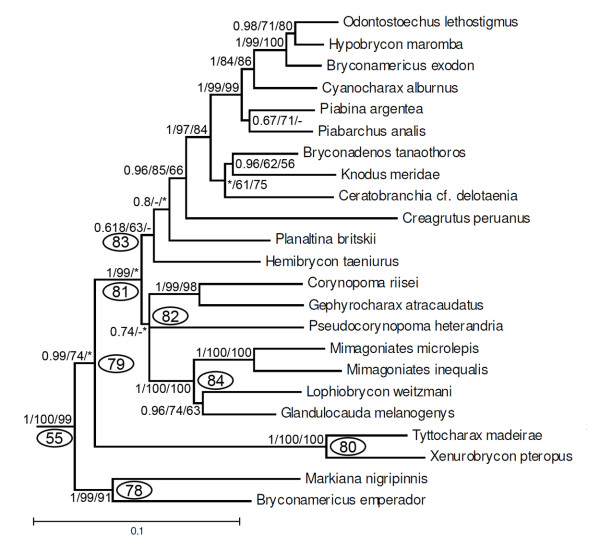
**Partial ML tree (see Figure 9 for overall view) showing relationships among species of the clade 55 (subfamily Stevardiinae)**. Numbered nodes as referenced in text and values as in Figure 4.

**Figure 13:**
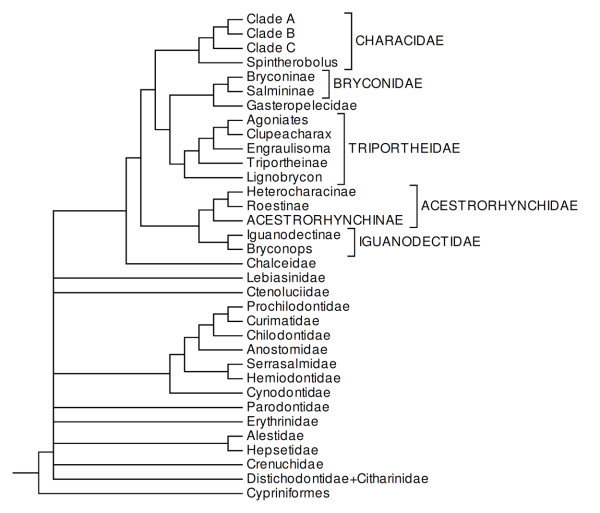
**Tree of the Characiformes with modified familial-level concepts indicated in uppercase**.

Throughout the text and in the figures, measures of support are indicated as a series of three numbers on selected internal branches of the trees subtending labelled clades, starting with posterior probabilities in Bayesian analysis (B) and followed by non-parametric bootstrap percentages from ML and MP analyses, respectively (e.g. 0.9/87/75, see Figure 4), dashes represent values lower than 0.5 (B) or 50% (ML, MP), and asterisks represent nodes that were not obtained by B or MP analyses. Nodes without support values greater than 0.5 (B) or 50% (ML, MP) were collapsed in all trees. A ML tree summarizing the phylogenetic results is presented in Figure 4. The general tree topology observed in all analyses was very similar, although statistical support was not strong for some nodes. An important difference between results of B and ML versus MP analyses was that under MP, the Neotropical Ctenoluciidae appeared as sister group of the African Hepsetidae (instead of sister to Lebiasinidae as in Figure 4), however this hypothesis has low statistical support in the MP analysis (Figure 5). Since the most highly resolved topology was obtained by ML analysis, this topology will be used to discuss relationships among taxa (Figures. 4, 6, 7, 8, 9, 10, 11, 12, 13), but important differences with results obtained by B and MP analyses will be discussed in the text. For convenience, clades discussed in the text also are labelled with numbers in the figures. Base composition was computed for all taxa on the concatenated alignment excluding constant sites to gauge the effect of possible base compositional bias on the resulting phylogeny. The percent of G+C (GC content) among 2885 variable sites was relatively homogeneous, ranging between 41.6% to 53.6%, but only few taxa had extreme values. For example, sequences with the lowest GC content (lower than 46%) were found among 12 taxa that are placed widely apart in the resulting phylogeny (e.g. *Phenagoniates, Catoprion, Crenuchus*). Likewise, sequences with the highest GC content (higher than 52%) were found in taxa such as *Cyanocharax, Xenocharax, Hollandichthys, Cheirodon, Cynopotamus*, and *Chalceus *that also are nested in widely separate clades. Hence, base compositional bias as a source of systematic error does not seem to affect the phylogenetic results.

### Phylogenetic relationships

Although a test of the monophyletic nature of the Characiformes was not the objective of the present study, rooting the tree in the Cypriniformes resulted in the Characiformes (clade 01) appearing as a well supported monophyletic group (values representing B posterior probability/MP bootstrap/ML bootstrap are: 1/100/87) in all analyses (Figure 4). Within the Characiformes, the African families Citharinidae and Distichodontidae (Figure 6, clade 02, 1/78/68) form the sister group to all remaining members of the order (clade 03). Although the clade formed by those two families has been found in all analyses, monophyly of the Distichodontidae was not supported by the data since *Citharinus *(Citharinidae) is embedded within that family (Figure 6).

Clade 03 (Figures 4, 6, 1/52/-) is composed by the Crenuchidae (clade 04) plus all remaining components of the Characiformes (clade 05). The monophyly of the Crenuchidae was strongly supported (Figure 6, 1/100/99) and the data show a clear separation of *Crenuchus *and *Poecilocharax*, subfamily Crenuchinae (1/100/100), from *Characidium *and *Melanocharacidium*, subfamily Characidiinae (1/100/100). *Characidium*, however, was not supported as a monophyletic group, since *Melanocharacidium *is nested within it (Figure 6).

Although clade 05 (Figure 6, 1/51/*) is not unanimously supported in all analyses, it contains a well-supported group (Figure 6, clade 06, 1/98/57) that includes the African families Hepsetidae (clade 08, a single specimen analyzed) and Alestidae (clade 09, 1/100/100). These two families are the sister group to the remaining taxa (clade 07, Figure 4, 0.89/-/*), but note that this topology only received marginal support from Bayesian analysis.

Clade 07 (Figure 4) is composed by two major groups, clades 10 (0.98/51/-) and 11 (0.97/-/*) none of which received unanimous support. Clade 10, however, is formed by several readily recognized and well-supported characiform families (Figures 4, 7). Within this clade, the Erythrinidae (clade 12, 1/99/100) is the sister group of all remaining members (Figure 7, clade 13, 1/88/63), and has *Hoplias *as the sister group of *Erythrinus *plus *Hoplerythrinus*. Among the remaining taxa in clade 13, the Parodontidae (clade 14, 1/100/100) form the sister group to clade 15 (1/100/90), a well supported group that includes the characid subfamily Cynodontinae (Figure 7, clade 16, 1/100/100), represented by all three of its recognized genera. Cynodontinae is the sister group to all remaining taxa in this clade (Figure 7, clade 17, 0.99/81/57). These remaining taxa within clade 17 are split into two well supported groups. One of these groups is clade 18 (Figure 7, 0.99/72/80) composed of the Hemiodontidae plus Serrasalmidae. The other group is the "Anostomoidea" (clade 19, 1/99/86), previously obtained by Vari [9,26], Buckup [8] and Calcagnotto *et al*. [32], and composed by the families Anostomidae, Chilodontidae, Prochilodontidae and Curimatidae, all of which receive unanimous support, however, their reciprocal interrelationship could not be solved herein.

The second group within clade 07 is clade 11 (Figures 4, 8, 0.97/-/*), obtained by ML, but only supported by a significant Bayesian probability. It is composed by the clade 29 (1/61/-) formed by the families Ctenoluciidae (clade 31, 1/98/99) and Lebiasinidae (clade 32, 1/100/100), and clade 30, that received strong support in all analyses (1/99/94). Within clade 30, the genus *Chalceus *(clade 33, 1/100/100) is the sister group of all remaining taxa (Figure 8, clade 34, 1/96/65).

Clade 34, although well supported (1/96/55), includes three monophyletic subunits, clades 35 (1/96/68), 36 (1/92/-), and 37(1/100/92) whose relationships were not resolved (Figure 4). Clade 35 is itself split into two well supported groups. One of them is clade 38 (1/100/97) composed by the Iguanodectinae and *Bryconops affinis*, and the other group is clade 39 (1/100/98) that contains the Acestrorhynchidae, Roestinae and a well supported group (clade 43, 1/97/94) with *Hoplocharax goethei, Heterocharax macrolepis *and *Gnathocharax steindachneri*. Some differences from the ML topology shown in Figures 4 and 8 were obtained by MP analysis, where *Gnathocharax *and *Hoplocharax *are sister taxa (Figure 5). In the Bayesian analysis, the Acestrorhynchidae appeared as the sister group of Roestinae (with low posterior probability = 0.71).

Clade 36 includes two monophyletic units (Figure 9, 1/92/-). Clade 44 (1/100/64) formed by representatives of the characid subfamilies Triportheinae (*Lignobrycon myersi *and *Triportheus*), Clupeacharacinae, and the Agoniatinae and the *incertae sedis *characid *Engraulisoma taeniatum*. Clade 45 (0.97/77/-) is formed by the distinctive family Gasteropelecidae (clade 46, 1/100/100) and clade 47 (1/100/100) by the characid subfamily Bryconinae plus *Salminus*.

Clade 37 is very well supported (1/100/92) and composed by four monophyletic groups. The first is the genus *Spintherobolus *(Figure 9, clade 50, 1/100/100). This is a striking phylogenetic placement, since *Spintherobolus *was until now considered a component of the characid subfamily Cheirodontinae, but the other putative members of this subfamily are nested within clade 77 (Figure 11), one of the groups nested in clade 53. *Spintherobolus *is the sister group of clade 51 (Figure 9, 1/89/-), composed by three monophyletic units, clade 52 that is the sister group of the monophyletic unit composed by clades 54 and 55 (Figure 9). Clade 52 (Figure 10, 1/100/100) includes elements of the subfamilies Stethaprioninae, Rhoadsiinae and species of other 29 genera such as *Gymnocorymbus, Nematobrycon, Moenkhausia*, and *Oligosarcus*. Within this group also are placed species of *Astyanax, Hemigrammus, Hyphessobrycon*, and *Jupiaba *but the monophyly of these genera is not supported by the results.

Within clade 54 (Figure 11, 1/68/*), *Exodon paradoxus *and *Roeboexodon guyanensis *form the sister group to two species of *Tetragonopterus *(clade 73), the only genus in the Tetragonopterinae, plus *Microschemobrycon casiquiare*, and five genera of the Characinae (clade 75). *Microschemobrycon casiquiare *appears as the sister group of all the Characinae (Figure 11, clade 74, 1/100/100). In all analyses, the monophyly of the Characinae was refuted due the association of *Hoplocharax, Heterocharax *and *Gnatocharax *(clade 43) previously assigned to this subfamily within clade 41 (Figure 8, discussed above). Within clade 70 are all examined representatives of the subfamily Cheirodontinae (clade 77, 1/100/100) except for *Spintherobolus *(as noted above), and clade 76 (1/100/96) that includes the three examined members of the subfamily Aphyocharacinae and the genera *Aphyocharacidium, Paragoniates, Phenagoniates, Xenagoniates, Leptagoniates *and *Prionobrama*, all presently considered *incertae sedis *in the Characidae.

The final group in clade 37 consists of what has most recently be considered to be the Stevardiinae according to Mirande [23]; albeit with the addition of *Markiana nigripinnis *which that author has as part of the *Astyanax *clade within the Characidae. These results differ from those of the earlier study by Weitzman *et al*. [20] under which the members of their more restricted Stevardiinae fall into different subunits of clade 55 (Figure 12). These are clade 80 (1/100/100) including *Tyttocharax madeirae *and *Xenurobrycon pteropus*, part of clade 82 formed by *Corynopoma riisei, Gephyrocharax atracaudatus, Pseudocorynopoma heterandria*, and by *Planaltina *which is located inside clade 83. The Stevardiinae of Weitzman *et al*. [20] additionally differs from clade 55 in not including representatives of *Bryconamericus, Bryconadenos, Ceratobranchia, Creagrutus, Cyanocharax, Hemibrycon, Hypobrycon, Knodus, Odontostoechus, Piabarchus*, and *Piabina *all of which were considered *incertae sedis *in the Characidae by Lima *et al*. [29]. In the analysis, *Bryconamericus emperador *does not form a monophyletic group with *B. exodon*, the type species of this genus, a result indicating the non-monophyly of the genus.

Some key alternative hypotheses were tested to assess their support in light of the new molecular evidence. The topologies derived from studies by Lucena and Menezes [13], Calcagnotto *et al*. [32], and Mirande [22,23] produced likelihood scores that are significantly worse than the score of the ML tree (lnL = - 159175.3 obtained with Treefinder), and are therefore rejected by the new data (Table 4). However, the ML tree (summarized in Figure 4) and several topologies with alternative branching patterns among at the basal-most nodes are statistically indistinguishable under maximum likelihood (Table 4). Ln Likelihood differences of up to 40 units from the ML tree score are not significantly rejected by the SH and AU tests. A set of nine topologies tested involve alternative placements among the following early-branching lineages of Characiformes: Distichodontidae + Citharinidae (clade 02), Crenuchidae (clade 04), Alestidae + Hepsetidae (clade 06), Parodontidae (clade 14), Erythrinidae (clade 12), Lebiasinidae (clade 32), Ctenoluciidae (clade31) and clade 15 (Cynodontidae, Anostomoidea, Serrasalmidae and Hemiodontidae). Although an exhaustive search of all possible trees with alternative branching patterns of these lineages was not performed, results from the tested topologies show that at least five of the nine trees cannot be rejected under this criterion. This result is consistent with the low support values obtained for clades 03, 05, 07, 10, 11, 13, and 29 (Figures 4, 5, 6, 7, 8). Therefore, the relative position of these groups remain unresolved in our study, as reflected in Figure 13. Interestingly, a topology that reflects reciprocal monophyly of African and Neotropical groups obtained a LnL score only 37.2 units worse from the ML score and is only marginally worse than the ML topology.

**Table 4:** Likelihood-based tests for alternative topologies

Topology^a^	Ln Likelihood	Diff.^b^	SH	AU
(1,(2,(3,((4,(5, 6)),((7, 8), R)))))	-159175.3	0.0	1.0000	0.9139
(7,(1,(2,(3,(4,((5, 6),(8, R)))))))	-159195.4	20.1	0.4269	0.2226
(1,(2,(7,(3,(4,((5, 6),(8, R)))))))	-159195.7	20.4	0.4109	0.0514
(1,(7,(2,(3,(4,((5, 6),(8, R)))))))	-159198.9	23.6	0.3037	0.0766
(7,(1,(2,(3,(6,(8,(5,(4, R))))))))	-159200.5	25.2	0.2912	0.1972
(7,(1,(3,(2,(4,((5, 6),(8, R)))))))	-159201.4	26.1	0.2531	0.0882
((1, 3),(7,(2,((5, 6), (8, R)))))^c^	-159212.5	37.2	0.1071	0.0000*
(7,(1,(2,(3,((4, 6),(5,(8, R))))))	-159215.6	40.3	0.0446*	0.0000*
(1,(2,(7,(3,(4,(5,(6,(8, R))))))))	-159219.3	44.0	0.0371*	0.0000*
(1,(4,(7,(2,(3,(6,(8,(5, R))))))))	-159220.9	45.6	0.0485*	0.0295
Calcagnotto *et al*. [31]	-159685.2	509.9	0.0145*	0.0000*
Lucena and Menezes [12]	-159692.4	517.1	0.0128*	0.0000*
Mirande [22], node 176 only	-160345.9	1170.6	0.0000*	0.0000*
Mirande [22]	-166637.0	7461.7	0.0000*	0.0000*

SH and AU are probability values obtained for the Shimodaira-Hasegawa and the Approximately Unbiased tests (Shimodaira 2002). Asterisks denote significant values (P < 0.05 for SH and P < 0.01 for AU), that imply the topology is rejected.

^a ^Topologies are sorted by likelihood values (obtained with Treefinder); at the top is the unconstrained ML topology summarized in Figure 4. Alternative hypotheses tested are depicted by parenthetic notation, where numbers represent the following taxa: 1: Distichodontidae+Citharinidae, 2: Crenuchidae, 3: Alestidae+Hepsetidae, 4: Erythrinidae, 5: Parodontidae, 6: Cynodontidae+Anostomoidea+Serrasalmidae+Hemiodontidae, 7: Ctenoluciidae, 8: Lebiasinidae, R: the rest (node 31 in Figure 4). Other topologies tested were taken from Calcagnotto *et al*. (2005); Lucena and Menezes (1998): the monophyly of Cynodontidae according to these authors groups: *Cynodon, Hydrolycus, Rhaphiodon, Gilbertolus*, and *Roestes*; Mirande (2010): either his full cladogram or only a constraint to impose his node 176 (Salmininae, Agoniatinae, Acestrorhynchinae, Cynodontidae).

^b ^Difference in Ln likelihood score with the best tree and the alternative topologies tested.

^c ^This topology is consistent with the reciprocal monophyly of African and Neotropical species.

## Discussion

Although many important studies have been conducted to infer relationships among families in the Characiformes at different levels, this problem was previously only partially tested because of incomplete sampling across the order [8,22,23,32,59] or due to a lack of resolution in the data [30]. But some consensus seems to be emerging [60]. The studies of Ortí and Meyer [30], Buckup [8], Calcagnotto *et al*. [32] and the present results (Figures 4, 6) corroborate the hypothesis of Vari [25] that the Citharinidae and Distichodontidae form a monophyletic lineage and are the sister group to all other Characiformes. Topology tests suggest that alternative placement of this lineage cannot be rejected, and our data suggest that some components of the Distichodontidae (*Distichodus*) may be more closely related to the Citharinidae (*Citharinus*) than to other taxa now assigned to the Distichodontidae (Figure 6). This conclusion runs counter to the morphological evidence, in particular the very unusual mobile hinge between the dentary and more posterior components of the lower jaw [24] and should be further tested using additional taxa and molecular characters.

The second lineage observed in our results is the family Crenuchidae (Figures 4, 6). Up to this time, the position of the Crenuchidae among characiforms has been problematic (Figures 1, 2b) with different authors suggesting alternative phylogenetic placements [8,23,30,32]. Even though our results suggest that this group forms one of the early branching events within the order, support for this position is not strong and should be considered provisional. Only five taxa of the Crenuchidae were included in this analysis (Figure 6), but the monophyly and distinctiveness of these taxa received strong support in our study, as does its division into the subfamilies Crenuchinae and Characidiinae as proposed by Buckup [8]. On the other hand, the two analyzed species of *Characidium *did not appear as sister taxa, suggesting the genus may be polyphyletic (Figure 6) a hypothesis that should be tested in the future with additional data.

Results based on Bayesian and ML analyses strongly suggest a close relationship between the Alestidae and Hepsetidae (clade 06, Figures 4, 6), a conclusion at variance with hypotheses proposed by Ortí and Meyer [30], Buckup [8], Calcagnotto *et al*. [32], and Zanata and Vari [5]. In our MP analysis the Hepsetidae appears, however, as the sister group of the Ctenoluciidae as proposed by Buckup [8]. Unfortunately, the backbone nodes in the phylogeny separating Hepsetidae and Ctenoluciidae (Figure 4) are all weakly supported by ML and MP analyses, and the topology tests suggest that alternative placements of this lineage are not significantly different (Table 4). Interestingly, our study (in agreement with Calcagnotto *et al*. [32], and Arroyave and Stiassny [61]) indicates that the Alestidae is not the sister group of *Chalceus *as proposed by Zanata and Vari [5] and Mirande [22,23] based on morphological evidence.

All of the analyses in this study strongly support a large Neotropical clade composed by the "Anostomoidea" (Anostomidae, Chilodontidae, Curimatidae, Prochilodontidae), plus Cynodontinae, Serrasalmidae, Hemiodontidae (Figure 7, clade 15, 1/100/90). Although other authors suggested a close relationship among some of these families [8,9,30,32] the final composition and arrangement of this group arrived at in this study represents a novel hypothesis of relationship among these taxa. In some of our results, the Erythrinidae appears as the sister group of clade 13, (albeit with low support especially by ML and MP criteria), a hypothesis at variance with morphologically based studies which associated the family in varying combinations with the Ctenoluciidae, Hepsetidae and Lebiasinidae [62]. In contrast, Calcagnotto *et al*. [32] obtained support for a sister group relationship between the Erythrinidae and Crenuchidae. If supported by future studies our hypothesis would indicate that many of the apparent morphological synapomorphies among the Ctenoluciidae, Hepsetidae and Lebiasinidae may be convergences perhaps associated with modifications necessary for predatory life styles. At this stage, however, relationships among these early branching lineages of characiforms (Erythrinidae, Crenuchidae, Ctenoluciidae, Alestidae, Hepsetidae, Lebiasinidae, Parodontidae, and clade 15) remain poorly resolved. Our results, however, indicate that within the Erythrinidae, *Erythrinus *and *Hoplerythrinus *form a monophyletic group which is the sister group of *Hoplias*. This is the first published hypothesis of relationship among genera of the Erythrinidae and should be tested in further studies in this family.

The monophyly of Cynodontidae, as proposed by Lucena and Menezes [13], is rejected by our phylogenetic analysis and topology tests (Figure 6 and Table 4); instead, representatives of Roestinae (*Roestes *and *Gilbertolus*) are placed more closely related to the Acestrorhynchidae (Figure 8). The monophyly of the Cynodontinae as proposed by Toledo-Piza [17] was, however, corroborated but internal relationships among the genera of the Cynodontinae found in the present study differ, since herein we find *Rhaphiodon *and *Hydrolycus *as sister groups while Toledo-Piza [17] proposed *Rhaphiodon *as the sister group of *Cynodon*.

A sister group relationship between the Hemiodontidae and Serrasalmidae (clade 18, Figure 7) has never been proposed. Calcagnotto *et al*. [32], however, hypothesized that the Serrasalmidae forms the sister group of a clade consisting of five Neotropical families, one of which was the Hemiodontidae (Figure 1). The monophyly of the Hemiodontidae as proposed by Langeani [27] is corroborated. The piranhas and pacus form a distinct and strongly monophyletic group, historically considered a subunit of the Characidae; however, the distinctive external anatomy of the members of the serrasalmids [63] and its phylogenetic separation from other taxa typically associated with the Characidae lead various authors to consider this group as a family distinct from the Characidae (e.g., Ortí *et al*. [7]). Based on the arrived at scheme of relationships, and in order to keep the family Characidae monophyletic, we also recognize the family Serrasalmidae as valid. Although some genera and many species of the Serrasalmidae were not included in the present study, phylogenetic relationships within the family are in agreement with more detailed studies (e.g., Ortí *et al*. [7]).

The large assemblage composed by the Anostomidae, Chilodontidae, Prochilodontidae and Curimatidae supported in our results (Figures 4, 7) is similar to that obtained by Vari [9] and corroborated by Buckup [8] based on data from Vari [9]. In light of an extensive series of unusual modifications of the gill arches and anterior portion of the vertebral column, Vari [9] found a close relationship between the Chilodontidae and Anostomidae. Herein, the position of the Chilodontidae was unresolved, thus, the hypothesis of Vari [9] about the pattern of relationships among these families should be tested in the future with the inclusion of more data and taxa.

### Monophyly and composition of Characidae

At present two conflicting hypothesis are available regarding the family Characidae. A broad concept is that employed by Nelson [1] and Reis *et al*. [6] in which 12 subfamilies and about a hundred *incertae sedis *genera are included in this family. This concept is very close to that proposed by Mirande [22,23] that differs from the schemes of previous authors by the exclusion of Serrasalmidae from, and inclusion of the Acestrorhynchidae and Cynodontidae in, the Characidae (Figure 2). According to Mirande [23] this broad Characidae is characterized by eight synapomorphies, only one of which, the fusion of the anteriormost procurrent caudal-fin rays into medial bony plates running parallel to the remaining rays (character 305), is an unreversed and uncontradicted synapomorphy of the Characidae. A more restricted concept of the Characidae is that proposed by Malabarba and Weitzman [19] according to whom this family is composed only by characiform species lacking the supraorbital bone (Figure 2). This restricted group is recognized by Mirande [22,23] who proposed that the absence of a supraorbital bone characterizes a monophyletic group of "distal" characids.

The present results could be interpreted in alternative modes taxonomically but in order to maintain the previously recognized families Cynodontidae, Acestrorhynchidae and Gasteropelecidae and in order to simplify the recognition of the family Characidae we suggest usage of a more restricted composition for this family, as described below.

Clade 30, a robust result in all analyses (Figures 4, 8), encompasses all species currently grouped in the Characidae (*sensu *Reis *et al*. [6]), with the exception of the Serrasalmidae but with the addition of the Gasteropelecidae, Acestrorhynchidae and Roestinae (a subfamily of the Cynodontidae *sensu *Lucena and Menezes [13]) (Figures 4, 5, 8). The genus *Chalceus*, previously placed with Alestidae by Zanata and Vari [5] and Mirande [22,23] based on morphological features, was found here as the basal branch in clade 30, a hypothesis also congruent with results of Calcagnotto *et al*. [32]. Since *Chalceus *belongs to an important monophyletic lineage either as the sister group of a large assemblage of Neotropical characids, as proposed herein, or closely related to the Alestidae as suggested by the morphological analysis [5] we recognize the family Chalceidae in the sense of Albert *et al*. [64] consisting of the species of *Chalceus*, to highlight this phylogenetically significant monophyletic group (Figure 13).

Clade 35 (Figure 8) contains the representatives of Acestrorhynchidae, the subfamilies Iguanodectinae and Roestinae, some genera of Characinae and *Bryconops*. The Iguanodectinae (two valid genera) is placed as the sister group of *Bryconops affinis *(Figure 8). Mirande [23] also found a monophyletic Iguanodectinae closely related to a named '*Bryconops' *clade. In order to recognize this monophyletic assemblage within the Characiformes, we propose that *Iguanodectes, Piabucus *and *Bryconops *be united in the family Iguanodectidae (Figure 13).

Acestrorhynchidae is placed as the sister group of the Roestinae, a subfamily that is presently assigned to the Cynodontidae, along with some species of the Characinae (*Gnathocharax, Heterocharax, Hoplocharax*) (Figure 8). A close relationship between the Acestrorhynchidae and Roestinae was previously proposed by Lucena and Menezes [13]. Alternatively, Malabarba and Weitzman [19] questioned the relationship between the Roestinae and Cynodontinae because of the presence in adults of *Gilbertolus *and *Roestes *of bony hooks on the fin rays versus the absence of those elaborations in the fins of *Cynodon, Rhaphiodon *and *Hydrolycus*. Our results indicate that the Cynodontinae and Roestinae are not closely related, refuting the monophyly of the Cynodontidae of Lucena and Menezes [13]. Mirande [22] found a monophyletic group composed by *Acestrorhynchus pantaneiro *and *Rhaphiodon vulpinus *(the only species of the Cynodontidae analyzed in that study), a hypothesis which is refuted in the present study since *Rhaphiodon *and the other two genera of the Cynodontinae, are grouped with high support in clade 10 (Figure 7). Lucena [65] was the first author to propose the monophyly of a clade composed of *Gnathocharax, Heterocharax, Hoplocharax *and *Lonchogenys*. Mirande [22] found the same monophyletic group and proposed the subfamily Heterocharacinae to contain *Gnathocharax *(not studied by that author), *Heterocharax, Hoplocharax *and *Lonchogenys*. Although representatives of *Lonchogenys *were not analyzed in the present study, a monophyletic group formed by *Gnathocharax, Heterocharax *and *Hoplocharax *also was obtained in our results (clade 43, Figures 4, 5, 8). The genera *Gnathocharax, Heterocharax, Hoplocharax *and *Lonchogenys *were assigned to the Characinae by Lucena and Menezes [58] but differ from the other members of this subfamily in several synapomorphies [22,23]. Therefore, we suggest the retention of the Heterocharacinae (*sensu *Mirande [22]) to refer to taxa in clade 43. In order to delimit our clade 39 as monophyletic unit within characiforms we propose that the Acestrorhynchidae as currently defined [66] be ranked as a subfamily (Acestrorhynchinae); and the group composed by the Roestinae, Heterocharacinae (*sensu *[22]) and Acestrorhynchinae, as newly defined, form a more encompassing Acestrorhynchidae (Figure 13).

Another well supported group of note is clade 36 in which the Agoniatinae (both of the recognized species included in our analysis) forms a monophyletic lineage with the Clupeacharacinae (only one recognized species) and the group formed by these taxa is the sister group of *Engraulisoma *(a single described species). This monophyletic clade is, in turn, the sister group of *Triportheus *(two of nineteen species included) in the B and ML analyses (Figure 9) or as the sister group of the Triportheinae in the MP analysis (Figure 5). In the B and ML analyses *Lignobrycon *(a single Recent species) is the sister group of all remaining taxa in the clade 44 (Figures 4, 9). Relationships across the spectrum of these groups were not previously investigated since earlier phylogenetic studies involving characiforms and/or characids included only a few representatives from this clade [22,23,30,32,33]. Malabarba [15] found eight synapomorphies supporting a close relationship between *Triportheus *and *Lignobrycon*, but it is uncertain whether the intervening taxa in the phylogeny arrived at herein were considered in her analysis. It would be informative to revisit the morphological hypothesis within the context of the results arrived at herein. In order to highlight this monophyletic lineage we expand the Triportheinae to include the genera *Agoniates, Clupeacharax, Triportheus, Engraulisoma*, and *Lignobrycon *and recognize it as the Triportheidae (Figure 13).

The Gasteropelecidae (clade 46) is placed as the sister group of the Bryconinae plus *Salminus *(clade 47, Figures 4, 5, 9), albeit without unanimous support. The three genera of the Gasteropelecidae formed a monophyletic group in which *Carnegiella *is the sister group of *Gasteropelecus *(Figure 9). In contrast, Javonillo *et al*. [33] found *Thoracocharax *to be the sister group of *Gasteropelecus *with *Carnegiella *the sister group of that clade. In his pre-cladistic analysis of the Gasteropelecidae, Weitzman [67] considered *Carnegiella *to be an evolutionary "development" of *Gasteropelecus*, mainly through structural losses. Subsequently Weitzman [68] stated that *Thoracocharax *has a "somewhat more primitive morphology with respect to other members of the Gasteropelecinae (= Gasteropelecidae)" and arose from a common ancestor with *Gasteropelecus*. He also proposed that *Carnegiella *"seems to be a neotenic form of *Gasteropelecus *and directly derived from it". Our results are, thus, congruent with the proposals of Weitzman [67,68] but not the hypothesis of Javonillo *et al*. [33].

A close relationship between *Brycon *and *Salminus *(Figure 9) was recognized by Géry [63] who suggested that the tribe Salminini was part of the Bryconinae. This hypothesis was corroborated in the phylogenetic studies of Ortí and Meyer [30], Calcagnotto *et al*. [32], and Javonillo *et al*. [33]. Calcagnotto *et al*. [32] found that *Salminus *was placed inside *Brycon *and Javonillo *et al*. [33] found *Salminus *to be the sister group of the two analyzed species of *Brycon*. Herein, the monophyly of *Brycon *was rejected since *B. insignis *appeared more closely related to *Henochilus wheatlandii *than it is to *B. amazonicus*. This would indicate that further studies are necessary to determine whether *Henochilus *must be synonymized with *Brycon I*. Mirande [22] previously proposed the subfamily Salmininae which is corroborated in the present study. To highlight the monophyletic group composed by the Bryconinae and Salmininae we expand the previous concept of the Bryconidae to include the Salmininae (Figure 13).

A strongly supported monophyletic group, clade 37, was observed in all our analysis including what we recognize as the family Characidae (Figures 9, 13). The composition of this group is largely comparable to the taxon of that name previously recognized by Malabarba and Weitzman [19] and is characterized morphologically by the lack of a supraorbital bone in its members, a synapomorphy of the Characidae as per this definition (Figure 2a, character 2). The sole difference between the Characidae of this study and that of Malabarba and Weitzman [19] is that under our results the Iguanodectinae is more closely related to *Bryconops *than it is to the taxa herein assigned to the Characidae (see discussion above).

An additional morphological character potentially supporting the hypothesis of the monophyly of clade 37 is the emergence of the hyoid artery from the anterior ceratohyal proximate to the articulation of that bone with the posterior ceratohyal. This feature noted by Castro [69], was more recently used by Mirande ([23]; character 178) as a synapomorphy of his node 204 which in that phylogeny included all characiforms without a supraorbital bone.

In all analyses *Spintherobolus *(clade 50) appears as sister group of all remaining characids in clade 37 (Figure 9). Three of the four recognized species of *Spintherobolus *were sequenced, excluding the type species (*S. papilliferus*). This result indicates that the subfamily Cheirodontinae as now defined [70] is not monophyletic in that the remaining members of the subfamily do not resolve as the sister group of *Spintherobolus *in this analysis. Recent investigations of the monophyly of the Cheirodontinae and the placement of the *Spintherobolus *involved only a subset of the members of the subfamily [22,23,33,71] and the results herein indicate that the question should be reinvestigated utilizing more complete intrasubfamilial representation.

The remaining species of the Characidae are included in three clades, each with large numbers of species: clade 52 (Figure 10), clade 54 (Figure 11), and clade 55 (Figure 12) in the Bayesian and ML analyses. Interestingly these three same clades, named clades C, B, and A, respectively, were found in the molecular phylogeny of Javonillo *et al*. [33] using sequences of some different genes. Clades 54 and 55 (Figure 9) also form a monophyletic group, as proposed by Javonillo *et al*. [33]. Clade 55 was recognized by Mirande [22,23] as a broad Stevardiinae which is partially in agreement with our results and this name was here applied to clade A. In light of the still evolving state of knowledge of many genera and species in the Characidae we prefer to not formally name the clades B, and C as proposed by Javonillo *et al*. [33].

Clade 52 (Figure 10) is equivalent to clade C of Javonillo *et al*. [33]. This is the most species-rich group within the Characiformes with more than 500 species [72]. This clade encompasses the speciose genera *Astyanax, Hemigrammus, Hyphessobrycon*, and *Jupiaba*, all of which are polyphyletic according to the results of this study and *Moenkhausia *which was previously demonstrated to be polyphyletic by Mirande [23] and Javonillo *et al*. [33]. Although we found several strongly supported groups discussed below, some basal nodes could not be resolved. Several groups with overall similar morphology and body shape were well supported, such as the group composed by *Hollandichthys, Rachoviscus *and *Bario *and the group consisting of *Ctenobrycon *plus *Psellogrammus*. An interesting and well supported group was formed by *Stygichthys typhlops*, a cave fish [73], and *Coptobrycon bilineatus*, as sister group of *Probolodus heterostomus, Deuterodon iguape *and *Myxiops aphos*. All of these species inhabit very ancient land formations in the northeastern and southeastern regions of Brazil, which are also the areas of residence of primitive lineages in other groups of fishes such as the Trichomycteridae [74] and Loricariidae [75].

Clade 52 has the four genera of Stethaprioninae as a monophyletic group, thereby corroborating the hypothesis advanced by Reis [12] based on morphological evidence. Reis [12] found that *Brachychalcinus *and *Stethaprion *are sister-groups to each other, *Orthospinus *is sister to that clade, and *Poptella *is the most basal genus in the group. The phylogenetic relationships found herein (Figure 10) differ from that hypothesis. We also found *Gymnocorymbus ternetzi *to be the sister group of the Stethaprioninae as proposed by Mirande [22,23]. The Rhoadsiinae, represented in our study only by *Carlana eigenmanni*, also falls within clade 52 and is hypothesized to be closely related to *Nematobrycon palmeri *(Figure 10), a result at variance with the hypothesis of Mirande [22,23].

The second of the large clades in the Characidae is clade 54 (Figure 11). A similar monophyletic group but represented by a smaller number of taxa was observed in the study by Javonillo *et al*. [33] and named clade B by those authors. This group is composed by two main lineages (clades 69 and 70). Within clade 69, clade 71 includes two genera, *Exodon *and *Roeboexodon*, now considered *incertae sedis *in the Characidae, as a monophyletic group that is sister to all remaining taxa in the clade. These species share a number of distinctive external features, most prominent among these being the mammiliform teeth external to the upper jaw. *Exodon *and *Roeboexodon *were grouped with *Bryconexodon *(not analyzed herein) in a monophyletic clade by Mirande [22] who hypothesized that the group was closely related to the Characinae. In contrast, our study has this group related to a larger clade composed of the Tetragonopterinae, *Microschemobrycon casiquiare*, and the Characinae (clade 72, Figure 11). A relationship of *Exodon *with the Tetragonopterinae and Characinae was also proposed by Javonillo *et al*. [33].

One of the interesting results in our study was the sister group relationship between *Microschemobrycon casiquiare *and the Characinae (clade 74, Figure 11). Mirande [22,23] found *Microschemobrycon *to be closely related to the other genera in his Aphyoditeinae, a group which had a composition similar to that initially proposed by Géry [63]. The Characinae in our results is similar to the group of that name as defined by Mirande [22,23] but as noted above, excluding *Exodon *and *Roeboexodon*. The Characinae of our study differs from that proposed by Lucena and Menezes [58] by the exclusion of *Gnathocharax, Hoplocharax *and *Heterocharax *(see discussion above). In our results, the two analyzed species of *Cynopotamus, C. kincaidi *and *C. venezuelae *do not form a monophyletic group thereby raising questions as to the monophyly of the genus. The Tetragonopterinae, restricted to the genus *Tetragonopterus *by Reis [76] is monophyletic and the sister group of the Characinae plus *Microschemobrycon*. Our data refutes the hypothesis of Mirande [23] who proposed a large Tetragonopterinae, including several genera which are not related to *Tetragonopterus *in the present study.

The second large group included in clade B is clade 70 (Figure 11) that is composed by the Aphyocharacinae and several genera currently considered *incertae sedis *in the Characidae. This group has a composition similar to that of the Aphyocharacinae plus Paragoniatinae of Géry [63] which was grouped by Mirande [22] in his redefined Aphyocharacinae (a subfamily that also includes *Inpaichthys *and *Rachoviscus *- not sampled in that study). The inclusion of *Rachoviscus *in the Aphyocharacinae was previously refuted by Thomaz *et al*. [77]. In our study, *Inpaichthys *and *Rachoviscus *belong to clade 52 (Figure 10) while *Aphyocharacidium bolivianum *appears as the sister group of all members of clade 76. Mirande [22,23], alternatively, suggested that *Aphyocharacidium *be included in his Aphyoditeinae. Relationships among these genera in our results differ notably from those proposed by Mirande [22]. In our results, the Aphyocharacinae appears as the sister group of *Prionobrama *and this group is, in turn, the sister group of the clade composed by *Paragoniates, Phenagoniates, Xenagoniates *and *Leptagoniates*. These last four genera share a very characteristic morphology with a very compressed, elongate body and a long anal fin [63].

The third large group in clade 54 is the subfamily Cheirodontinae (clade 77, Figure 11). As discussed above, however, the position of *Spintherobolus *at the base of the Characidae renders the Cheirodontinae *sensu *Malabarba [70] paraphyletic. The monophyly of the Cheirodontinae was previously supported by Calcagnotto *et al*. [32], Mirande [22] and Javonillo *et al*. [33] but without the analysis of a significant number of genera and species, most notably *Spintherobolus*. The division of the Cheirodontinae into the tribes Compsurini and Cheirodontini [14] was also not supported by the results of our analysis. Notably, we found that the trans-Andean cheirodontin species, *Nanocheirodon insignis *and *Pseudocheirodon arnoldi*, are the sister group of genera and species occurring in the cis-Andean region. This suggests a very old origin for this clade and more inclusive clades, predating the uplift of the northern Andean cordilleras.

Clade 55 (Figure 12) partially corresponds to clade A of Malabarba and Weitzman [19] who noted the similarity in composition of their clade A to a group proposed by Géry [63]. The monophyly of this clade was corroborated in the taxonomically broad study of Weitzman *et al*. [20] and Menezes and Weitzman [21], and in the analyses of Calcagnotto *et al*. [32] and Javonillo *et al*. [33], albeit based on a fewer number of analyzed taxa in these latter studies. Inside this group, we found a monophyletic Glandulocaudinae, *sensu *Menezes and Weitzman [21] (clade 84, Figure 12) in which *Glandulocauda *appears as the sister group of *Lophiobrycon *and with the clade formed by those taxa as the sister group of *Mimagoniates*. This is the first real test of the hypothesis of the monophyly of the Glandulocaudinae as delimited in recent studies, since only *Mimagoniates *was analyzed in previous studies [22,32,33]. This hypothesis differs from that of Castro *et al*. [78] and Menezes and Weitzman [21] who found *Glandulocauda *to be the sister group of *Mimagoniates *and the clade consisting of those taxa as the sister group of *Lophiobrycon*. An analysis incorporating additional species of *Glandulocauda *and particularly *Mimagoniates *is necessary to thoroughly investigate the relationships among these genera.

The second previous recognized characid subfamily found in clade 55 is the Stevardiinae, *sensu *Weitzman *et al*. [20]. Although only six of the seventeen recognized genera of the Stevardiinae were included in this study, the results indicate that this subfamily, as proposed by Weitzman *et al*. [20], is polyphyletic (Figure 12). *Gephyrocharax *and *Corynopoma *(tribe Corynopomini) are the sister group of *Pseudocorynopoma *(tribe Hysteronotini) with this group the sister group of the Glandulocaudinae. *Xenurobrycon *and *Tyttocharax *(tribe Xenurobryconini), appear as more basal clades within clade 79 while *Planaltina *(tribe Diapomini) appears as more derived but not closely related to the remaining analyzed species of the Stevardiinae, *sensu *Weitzman *et al*. [20].

The most basal group within clade 55 is composed of *Markiana nigripinnis *and *Bryconamericus emperador *(clade 78, Figure 12). The inclusion of *M. nigripinnis *within clade A is a novel hypothesis. Although this species has ii+9 dorsal-fin rays, the plesiomorphic condition according Malabarba and Weitzman [19], other morphological characteristics including spermatozoa ultrastructure which is very similar to those of the non-inseminating members of clade 55, the presence of only four teeth in the inner premaxillary tooth row, and a short triangular ectopterygoid which is never more than twice the length of the palatine [79] corroborate the hypothesis that *M. nigripinnis *belongs to clade 55. Mirande [22,23] did not find *Markiana *to be a member of clade A, but did propose a sister group relationship between *M. nigripinnis and Bryconamericus scleroparius*. Although only two species of *Bryconamericus *were included in the present study, the genus appears as polyphyletic since *B. exodon*, the type species of the genus, is hypothesized to be more closely related to *Odontostoechus *and *Hypobrycon *than to its nominal congener (Figure 12). A polyphyletic *Bryconamericus *was also obtained in the analyses by Mirande [22,23] and Javonillo *et al*. [33]; results emphasizing the need for a reappraisal of the limits of the genus. A monophyletic clade composed by *Odontostoechus, Hypobrycon *and some species of *Bryconamericus *and *Cyanocharax *was proposed by Javonillo *et al*. [33] and parallels the results of this study. The sister group of these genera in our analysis is the clade composed by *Piabina argentea *and *Piabarchus analis*. Our study is the first one to investigate the relationships of *Piabarchus *and this conclusion runs counter to the hypothesis of a sister group relationship between *Piabina *and *Creagrutus *proposed by Vari and Harold [18] and Mirande [22,23] based on morphological characters, but not that arrived at by Javonillo *et al*. [33] in their molecular analysis. A final monophyletic lineage within clade 55 is composed of *Knodus meridae *(the type species of the genus), *Bryconadenos tanaothoros*, and *Ceratobranchia cf. delotaenia *(Figure 12). A sister group relationship between *Knodus *and *Bryconadenos *was previously suggested by Weitzman *et al*. [20] and corroborated by Javonillo *et al*. [33]. Leaving aside differences in the included species, Mirande's [22] concept of the Stevardiinae is equivalent to clade 55 of this study other than for the addition of *Markiana nigripinnis*. As discussed previously the concept of the Stevardiinae proposed by Weitzman *et al*. [20] differs significantly from our results. We consequently recognize clade 55 as the Stevardiinae in the sense of Mirande [22] expanded to include *Markiana nigripinnis*.

## Conclusions

The definition of the Characidae (our clade 37) as proposed herein (Figure 13) is the most significant contribution of the present study, with both molecular (B = 1, ML = 100, MP = 92) and morphological evidence (lack of a supraorbital bone and emergence of the hyoid artery from the posterior portion of the anterior ceratohyal) supporting the recognition of a proposed monophyletic group encompassing approximately one-half of all Recent characiforms.

As noted in the introductory comments, the Characiformes in general and within that order, the Characidae in particular, are speciose assemblages encompassing a number of very distinctive taxa. The results of the present analysis of a large number of species including on the one hand representatives of all of the main lineages of the Characiformes and on the other a large dataset including genes with slow to moderate evolutionary rates provided insight into the phylogenetic relationships of a number of previously problematic taxa. Most notable among these were various genera previously placed as *incertae sedis *within the Characidae. These results demonstrate that this combination of large numbers of taxa and large datasets should be a productive method for future investigations of phylogenetic relationships among large groups such as the Characiformes. Such future analysis both within the Characiformes and in other groups will presumably provide insight as to the degree to which differences in results between studies with varying degree of taxonomic inclusiveness are a function of the absence of critical taxa versus inadequate datasets resulting in poor degrees of phylogenetic resolution. Notwithstanding the fact that the present study was based on multiple genes and the largest number of species to date in a molecular analysis of the Characiformes, future studies including additional genera and particularly species of species-rich genera are necessary to resolve the questions noted in the discussion and to further improve our understanding of phylogenetic relationships across the Characiformes.

## Authors' contributions

CO, RMCC participated equally in the design of the study. GSA, KTA and TCM did most of the laboratory experiments. CO, GSA, KTA, TCM and GO analyzed parts of the data and did phylogenetic analyses. All authors discussed results. CO, GO and RPV wrote substantial parts of the manuscript. All authors read and approved the final manuscript.

## Supplementary Material

Additional file 1 **Specimens used in the phylogenetic analysis**. Click here for file

Additional file 2 **Sequences of primers used in present study**. Click here for file

Additional file 3 **Species analyzed, collection number, specimen number, and GenBank accession numbers**. Click here for file
