# Supplementary material for: Phylogenetic relationships within the speciose family Characidae (Teleostei: Ostariophysi: Characiformes) based on multilocus analysis and extensive ingroup sampling
Source: BMC Evol Biol. 2011 Sep 26;11:275. doi: 10.1186/1471-2148-11-275 (PMC3190395; doi:10.1186/1471-2148-11-275)
Supplement: Additional file 1 — Specimens used in the phylogenetic analysis. [file 1471-2148-11-275-S1.DOC]

Additional file 1 - Specimens used in the phylogenetic analysis.

| Group/species | Voucher | Specimen | Locality | Latitude/longitude |
| --- | --- | --- | --- | --- |
| Characidae/Agoniatinae |  |  |  |  |
| *Agoniates anchovia* | LBP 6740 | 33471 | Lago Catalão/Manaus/AM/Brazil | S 03°09.761’ W 59°54.487’ |
| *Agoniates halecinus* | LBP 5503 | 26594 | Igarapé Uiratapuru/Laranjal do Jari/AP/Brazil | S 00°34’03’’ W 52°34’41’’ |
|  |  |  |  |  |
| Characidae/Aphyocharacinae |  |  |  |  |
| *Aphyocharax alburnus* | LBP 1587 | 11774 | Rio das Garças/Barra do Garça/MT/Brazil | S 15°54'18.1'' W 2°19'24.2'' |
| *Aphyocharax anisitsi* | LBP 3764 | 22190 | Rio Negro/Aquidauana/MS/Brazil | S 19°34'33.7' W 56°14'49.5'' |
| *Aphyocharax pusillus* | LBP 4046 | 22920 | Rio Moa/Cruzeiro do Sul/AC/Brazil | S 7°37'20.0'' W 2°47'42.2'' |
|  |  |  |  |  |
| Characidae/Bryconinae |  |  |  |  |
| *Brycon amazonicus* | LBP 2187 | 15565 | Laguna de Castilleros/Caicara del Orinoco/Bolivar/Venezuela | N 07º30’50,9’’ W 66º09’19,8’’ |
| *Brycon insignis* | LBP 2369 | 16075 | Lagoa Feia/Campos dos Goytacazes/RJ/Brazil | S 22°00' W 41°20' |
| *Henochilus wheatlandii* | LBP 1221 | 25846 | Rio Santo Antônio/São Sebastião do Rio Preto/MG/Brazil | - |
|  |  |  |  |  |
| Characidae/Characinae |  |  |  |  |
| *Acestrocephalus sardina* | LBP 6876 | 33172 | Rio Negro/São Gabriel da Cachoeira/AM/Brazil | S 00°08.156' W 67°05.057' |
| *Charax leticiae* | LBP 1480 | 12700 | Rio Taquari - Pesqueiro Recnato Alegre/Coxim/MS/Brazil | S 18°25'42.5'' W 54°50'02.8'' |
| *Cynopotamus kincaidi* | LBP 3225 | 19449 | Lagoa marginal/Nobres/MT/Brazil | S 14°40'32,8'' W 56°13'14,0'' |
| *Cynopotamus venezuelae* | LBP 6132 | 29515 | Rio Santa Rosa/Machiques de Perijá/Zulia/Venezuela | N 09°38'53.8' W 72°34'56.4'' |
| *Galeocharax knerii* | LBP 3496 | 20164 | Rio Tietê/Birigui/SP/Brazil | S 21°06'25.2'' W 50°15'52.7'' |
| *Gnathocharax steindachneri* | LBP 4496 | 24494 | Igarapé Puxirituba/Barcelos/AM/Brazil | S 00°53'18.6'' W 62°40'36/1'' |
| *Heterocharax macrolepis* | LBP 4494 | 24485 | Igarapé Puxirituba/Barcelos/AM/Brazil | S 00°53'18.6'' W 62°40'36/1'' |
| *Hoplocharax goethei* | LBP 4495 | 24489 | Igarapé Puxirituba/Barcelos/AM/Brazil | S 00°53'18.6'' W 62°40'36/1'' |
| *Phenacogaster calverti* | LBP 5582 | 27299 | Afluente Parnaíba/Santa Filomena/PI/Brazil | S 09°09'51’ W 45°51'15' |
| *Roeboides guatemalensis* | LBP 2755 | 18529 | Río Llano Sucio/Santa Rita Arriba/Colón/Panamá | N 09°19’26.2'' W 79°46'08.2'' |
|  |  |  |  |  |
| Characidae/Cheirodontinae |  |  |  |  |
| *Aphyocheirodon hemigrammus* | LBP 8306 | 40025 | Rio Araras/Araras/SP/Brazil | S 22°22'42.4' W 47°25'37.9'' |
| *Cheirodon killiani* | LBP 3115 | 19803 | Río La Laja/Monte Aguila/VIII Region/Chile | S 37°12'54.8'' W 72°26'49.1'' |
| *Cheirodon ibicuhiensis* | LBP 4777 | 25598 | Rio Guaíba/Barra do Ribeiro/RS/Brazil | S 30°18'03.9'' W 51°20'40.8'' |
| *Compsura heterura* | LBP 4733 | 24984 | Rio Ceará-Mirim/Natal/RN/Brazil | S 05°37'47'’ W 35°37'09'' |
| *Heterocheirodon yatai* | LBP 4872 | 24954 | Rio Yi/Durazno/Durazno/Uruguai | S 33°23'49'’ W 56°24'10'' |
| *Kolpotocheirodon theloura* | LBP 5033 | 25982 | Ribeirão Bananal/Distrito Federal/Brazil | S 15°43’42.7’ W 47°54’39.4'' |
| *Macropsobrycon uruguayanae* | LBP 6039 | 29061 | Rio Piquiri/Cachoeira do Sul/RS/Brazil | 30º14'46''S e 52º45'53''W |
| *Nanocheirodon insignis* | LBP 6104 | 27476 | Rio Apon Medio/Machiques de Perijá/Zulia/Venezuela | N 10°09'42.0' W 72°25'58.0' |
| *Odontostilbe sp.* | LBP 4650 | 22626 | Rio Araquá/Botucatu/SP/Brazil | S 22°47.135’ W 48°28.892 |
| *Odontostilbe fugitiva* | LBP 4052 | 22932 | Rio Moa/Cruzeiro do Sul/AC/Brazil | S 7°37'20.0'' W 72°47'42.2'' |
| *Prodontocharax melanotus* | AMNH | 233264 |  |  |
| *Pseudocheirodon arnoldi* | STRI | 5 |  |  |
| *Saccoderma melanostigma* | LBP 6103 | 27475 | Rio Apon Medio/Machiques de Perijá/Zulia/Venezuela | N 10°09'42.0' W 72°25'58.0'' |
| *Serrapinnus calliurus* | LBP 3731 | 22121 | Lagoa Marginal Rio Negro/Aquidauana/MS/Brazil | S 19°34'54.6' W 56°15'16.5'' |
| *Serrapinnus heterodon* | LBP 9039 | 37551 | Córrego Cachoeira | S 17°08'54.9' W 43°49'32.3'' |
| *Serrapinnus piaba* | LBP 8972 | 41813 | Córrego da Mata/Pedro Leopoldo/MG/Brazil | S 19°37'59.7'' W 44°06'25.5'' |
| *Spintherobolus ankoseion* | LBP 4725 | 24957 | Arroio que desaguá no lago Acaraí/São Francisco do Sul/SC/Brazil | S 26°17'35'’ W 48°35'21'' |
| *Spintherobolus broccae* | LBP 3916 | 22558 | Riacho sem nome afluente do rio Vermelho/Bertioga/SP/Brazil | S 23°46'16,2'' W 46°00'37,2'' |
| *Spintherobolus leptoura* | LBP 7544 | 36098 | Afluente rio Mumuna/Iguape/SP/Brazil | S 24°42'57.8'' W 47°41'28.3'' |
| Gen. & sp. nov. | LBP 5699 | 27603 | Córrego Taquaral/Barra do Garças/MT/Brazil | S 15°40.678' W 52°17.863'' |
|  |  |  |  |  |
| Characidae/Clupeacharacinae |  |  |  |  |
| *Clupeacharax anchoveoides* | LBP 5046 | 26012 | Lagoa Bairro Caiçara/Cáceres/MT/Brazil | S 16°06’56’ W 57°44’33’ |
|  |  |  |  |  |
| Characidae/Glandulocaudinae |  |  |  |  |
| *Glandulocauda melanogenys* | LBP 4507 | 24538 | Rio Paranapiacaba/Santo André/SP/Brazil | S 23°46'13.2'' W 46°18'39.6'' |
| *Lophiobrycon weitzmani* | LBP 1225 | 38090 | Rio Claro/Delfinópolis/MG/Brazil | S 20°20'32.2' W 46°47'12.2'' |
| *Mimagoniates inequalis* | LBP 3383 | 21274 | Arroio dos Corrientes/Pelotas/RS/Brazil | S 31°28'46.3'' W 52°12'46.9'' |
| *Mimagoniates microlepis* | LBP 1225 | 11077 | Bertioga/SP/Brazil | S 23°57,769' W 46°10,625' |
|  |  |  |  |  |
| Characidae/Iguanodectinae |  |  |  |  |
| *Iguanodectes geisleri* | LBP 4266 | 23840 | Igarapé Boiboi/Barcelos/AM/Brazil | 00°49'43.7'' W 62°49'59.8'' |
| *Piabucus melanostomus* | LBP 5109 | 26150 | Lagoa Bairro Caiçara/Cáceres/MT/Brazil | S 16°06'66'' W 57°44'33'' |
|  |  |  |  |  |
| Characidae/Rhoadsiinae |  |  |  |  |
| *Carlana eigenmanni* | LBP 3300 | 19864 | Rio Mandinga/Panama | - |
| *Carlana eigenmanni* | LBP 3301 | 19865 | Rio Playon Chico/Panama | - |
|  |  |  |  |  |
| Characidae/Stethaprioninae |  |  |  |  |
| *Brachychalcinus copei* | LBP 192 | 8853 | Igarapé São Francisco/Rio Branco/AC/Brazil | S 9°56,271’ W 67°52,923’ |
| *Orthospinus franciscensis* | LBP 8105 | 37555 | Rio Verde Grande/Jaíba/MG/ Brazil | S 15°19'24.2' W 43°39'52.5'' |
| *Poptella paraguayensis* | LBP 3732 | 21986 | Lagoa Marginal Rio Negro/Aquidauana/MS/Brazil | S 19°34'54.6' W 56°15'16.5'' |
| *Stethaprion crenatum* | LBP 4078 | 22994 | Rio Japiim/Mâncio Lima/AC/Brazil | S 07°34'28.8' W 72°55'24.9'' |
|  |  |  |  |  |
| Characidae/Stevardiinae |  |  |  |  |
| *Corynopoma riisei*^2^ |  |  |  |  |
| *Gephyrocharax atracaudatus* | LBP 2753 | 18519 | Río Llano Sucio/Santa Rita Arriba/Colón/Panamá | N 09°19’26.2'' W 79°46'08.2'' |
| *Planaltina britskii* | LBP 2598 | 17243 | Córrego Boa Vista dos Castilhos/Miraluz/SP/Brazil | S 21º00'46.6'' W 49º41'25.1'' |
| *Pseudocorynopoma heterandria* | LBP 2862 | 18570 | Rio Fau/Miracatu/SP/Brazil | S 24°12,441' W 47°28,616' |
| *Tyttocharax madeirae* | LBP 5145 | 33166 | Rio Japiim/Mâncio Lima/AC/Brazil | S 07°34'28.8' W 72°55'24.9'' |
| *Xenurobrycon pteropus* | LBP 9054 | 42218 | Mutumparaná/Mutumparaná/RO/Brazil | S 09°36'39.5'' W 64°55'38.9'' |
|  |  |  |  |  |
| Characidae/Tetragonopterinae |  |  |  |  |
| *Tetragonopterus argenteus* | LBP 3758 | 22029 | Rio Negro/Aquidauana/MS/ Brazil | S 19°34'33.7' W 56°14'49.5'' |
| *Tetragonopterus chalceus* | LBP 8268 | 37556 | Rio Verde Grande/Jaíba/MG/ Brazil | S 15°19'24.2' W 43°39'52.5'' |
|  |  |  |  |  |
| Characidae/Tripotheinae |  |  |  |  |
| *Lignobrycon myersi* | LBP 8094 | 37519 | Rio do Braço/Ihéus/BA/Brazil | S 14°41'11.7' W 39°16'28.0'' |
| *Triportheus nematurus* | LBP 39 | 3503 | Rio Miranda/Corumbá/MS/Brazil | S 19°34,630' W 57°01,123' |
| *Triportheus orinocensis* | LBP 2663 | 15580 | Laguna de Castilleros/Caicara del Orinoco /Bolivar/Venezuela | N 07º30’50,9’’ W 66º09’19,8’’ |
|  |  |  |  |  |
| Characidae *incertae sedis* |  |  |  |  |
| *Aphyocharacidium bolivianum* | LBP 9055 | 42219 | Arara/Arara/RO/Brazil | S 09°36'39.5'' W 64°55'38.9'' |
| *Aphyodite grammica* | LBP 9050 | 42214 | Rio Madeira/Pacaás/ Mutumparaná/ RO/Brazil | S 09°37'05.3'' W 64°56'01.3'' |
| *Astyanacinus moorii* | LBP 5783 | 28195 | Rio Muzambinho/Muzambinho/ MG/Brazil | S 21°21'03.1'' W 46°29'33.2'' |
| *Astyanax aeneus* | LBP 8938 | 42019 | Chichancanab lagoon/Quintana Roo/México | 19,882999420166/-88,8710021972656 |
| *Astyanax jordani* | LBP 4527 | 24599 | Aquarium/Brazil |  |
| *Astyanax mexicanus* | LBP 8937 | 42016 | Ojo San Bernabe/Spring/Nuevo Leon/México |  |
| *Bario steindachneri* | LBP 4389 | 24187 | Rio Branco/Porto Velho/RO/ Brazil | S 09º34'10.7'' W 63º58'08.2'' |
| *Bramocharax baileyi* | LBP 8940 | 42025 | Chisec/Chajmaic/Alta Verapaz/Guatemala | 15,72 -89,94 |
| *Bramocharax caballeroi* | LBP 8939 | 42022 | Catemaco/Veracruz/México | 18,877 -95,292 |
| *Bryconadenos tanaothoros*^2^ | MCP 40399 |  |  |  |
| *Bryconamericus emperador* | LBP 2754 | 18528 | Río Llano Sucio/Santa Rita Arriba/Colón/Panamá | N 09°19’26.2'' W 79°46'08.2'' |
| *Bryconamericus exodon* | LBP 7123 | 34200 | Córrego Lageadinho/Sapopema/ PR/Brazil | S 23°55'29.0'' W 50°37'27.3'' |
| *Bryconella pallidifrons* | LBP 4646 | 24696 | Aquarium |  |
| *Bryconops affinis* | LBP 262 | 4168 | Represa de Três Marias/Três Marias/MG/Brazil | S 18°13,661’ W 45°14,857’ |
| *Ceratobranchia cf. delotaenia* | LBP 3257 | 20042 | Rio Chontabamba/Oxapampa/ Pasco/Peru | S 10º36’06,6’’ W 075º29’10,8’’ |
| *Chalceus epakros* | LBP 5443 | 26504 | Rio Jari/Almeirim/PA/Brazil | S 00°38’46’’ W 52°30’33’’ |
| *Chalceus erythrurus* | LBP 4211 | 22727 | Rio Juruá/Cruzeiro do Sul/AC/Brazil | S 07°09'49.6' W 73°43'29.7'' |
| *Coptobrycon bilineatus* | LBP 3809 | 33169 | Afluente rio Itatinga/Bertioga/SP/Brazil | S 23°45'01.2' W 46°09'52.9'' |
| *Creagrutus peruanus* | LBP 3267 | 20057 | Rio Santa Cruz/Pozuzo/Pasco/Peru | S 10º02’20,4’’ W 075º34’55,4’’ |
| *Ctenobrycon hauxwellianus* | LBP 4095 | 23538 | Rio Japiim/Mâncio Lima/AC/Brazil | S 07°34'28.8' W 72°55'24.9'' |
| *Cyanocharax alburnus* | LBP 4746 | 25516 | Rio Guaíba/Barra do Ribeiro/RS/Brazil | S 30°17'07.0'' W 51°18'01.1'' |
| *Deuterodon iguape* | LBP 6827 | 33065 | Rio Fau/Miracatu/SP/Brazil | S 24°12,441' W 47°28,616' |
| *Engraulisoma taeniatum* | LBP 4038 | 22896 | Rio Moa/Cruzeiro do Sul/AC/ Brazil | S 7°37'20.0'' W 72°47'42.2'' |
| *Engraulisoma taeniatum* | LBP 4038 | 22897 | Rio Moa/Cruzeiro do Sul/AC/ Brazil | S 7°37'20.0'' W 72°47'42.2'' |
| *Exodon paradoxus* | LBP 4006 | 23040 | Lago Morto/São Félix do Araguaia/MT/Brazil | S 11°40'9'' W 50°51'0.30'' |
| *Gymnocorymbus ternetzi* | LBP 3737 | 21989 | Lagoa Marginal Rio Negro/ Aquidauana/MS/Brazil | S 19°34'54.6' W 56°15'16.5'' |
| *Hasemania sp.* | LBP5967 | 28455 | Rio Paraibuna/Comendador Levy Gasparian/RJ/Brazil | S 22°01'24.3'' W 43°10'08.5'' |
| Gen. and sp. new | LBP 7243 | 33196 | Rio Uberaba/Ponte Alta/MG/ Brazil | S 19°40'59.8'' W 48°40'08.6'' |
| *Hemibrycon taeniurus* | LBP 6847 | 33168 | Upper Arouca River/Trinidad Tobago | N 10°41.320' W 61°19.499' |
| *Hemigrammus marginatus* | LBP 6292 | 29419 | Córrego Barbacena/Pontal/SP/ Brazil | S 20°56'49.5'' W 48°08'51.9'' |
| *Hemigrammus ulreyi* | LBP 7604 | 36267 | Lagoa Margina rio Cuiabá/ Barão de Melgaço/MT/Brazil | S 16°11'39.5'' W 55°48'25.1'' |
| *Hollandichthys multifasciatus* | LBP 698 | 8791 | Afluente do rio Grande/ Paranapiacaba, SP/Brazil | S 23°46.123’ W 46°19.467’ |
| *Hyphessobrycon eques* | LBP 7615 | 36278 | Lagoa Margina rio Cuiabá/ Barão de Melgaço/MT/Brazil | S 16°11'39.5'' W 55°48'25.1'' |
| *Hyphessobrycon megalopterus* | LBP 7613 | 36932 | Lagoa Margina rio Cuiabá/ Barão de Melgaço/MT/Brazil | S 16°11'39.5'' W 55°48'25.1'' |
| *Hyphessobrycon reticulatus* | LBP 1049 | 8939 | Afluente rio São João/ Papandu/ SC/Brazil | S 26°22.049’ W 50°07.149’ |
| *Hypobrycon maromba* | LBP 6750 | 33174 | Rio Marombas/Curitibanos/ SC/Brazil | S 27°19’49,6’’ W 50°45’05,4’’ |
| *Inpaichthys kerri* | LBP 4526 | 24597 | Aquarium/Brazil |  |
| *Jupiaba anteroides* | LBP 7067 | 34380 | Igarapé Miuá/São Gabriel da Cachoeira/AM/Brazil | S 00°06.308' W 66°52.585' |
| *Jupiaba cf. acanthogaster* | LBP 7935 | 37269 | Rio dos Patos/Nova Mutum/MT/Brazil | S 13°48'03.1'' W 56°01'38.4'' |
| *Knodus meridae* | LBP 7569 | 15818 | Rio Orinoco/Caicara del Orinoco /Bolivar/Venezuela | N 07º39’06,3’’ W 66º10’34,2’’ |
| *Leptagoniates steindachneri* | LBP 4137 | 23661 | Rio Moa/Mâncio Lima/AC/Brazil | S 07°26'35.5' W 73°03'33.5'' |
| *Markiana nigripinnis* | LBP 663 | 8038 | Região de Rombado, afluente rio Pirai/Poconé/MT/Brazil | S 16°25,680' W 56°25,143' |
| *Microschemobrycon casiquiare* | LBP 8161 | 38058 | Rio Tapajós/Pimental/PA/Brazil | S 04°32'25'' W 56°15'15'' |
| *Moenkhausia xinguensis* | LBP 6101 | 28443 | Rio Culuene/Paranatinga/ MT/Brazil | S 13°49'00.0'' W 53°15'00.0'' |
| *Myxiops aphos* | LBP 7184 | 33173 | Rio Lençóis/Lençóis/BA/Brazil | S 12°33'41.8'' W 41°24'09.3'' |
| *Nematobrycon palmeri* | LBP 6124 | 33165 | Aquarium/Brazil |  |
| *Nematocharax venustus* | LBP 8106 | 37557 | Rio Almada/Ilhéus/BA/Brazil | S 14°39'52.1' W 39°13'26.7'' |
| *Odontostoechus lethostigmus* | LBP 6752 | 33171 | Arroio Água Parada/Maquiné/ RS/Brazil | 29°39’42,8’’S / 50°12’37,7’’ |
| *Oligosarcus paranensis* | LBP 3926 | 22582 | Rio Paraitinguinha/Salesópolis/ SP/Brazil | S 23°31'25.6'' W 43°53'22.7'' |
| *Oligosarcus hepsetus* | LBP 2377 | 16055 | Lagoa Feia/Campos dos Goytacazes/RJ/Brazil | S 22°00' W 41°20' |
| *Paracheirodon axelrodi* | LBP 4472 | 24425 | Igarapé Zalala/Barcelos/ AM/Brazil | S 00°40'03.1'' W 62°58'23.5'' |
| *Paragoniates alburnus* | LBP 9208 | 43156 | Rio Manapire/Cabruta/ Guárico/Venezuela | N 7°52'04.1" W 66°12'40.1" |
| *Parecbasis cyclolepis* | LBP 9053 | 42217 | Belmont – Foz/Belmont/RO/ Brazil | S 08°37'17.2'' W 63°49'25.7'' |
| *Phenagoniates macrolepis* | LBP 6105 | 35623 | Rio Apon Medio/Machiques de Perijá/Zulia/Venezuela | N 10°09'42.0' W 72°25'58.0'' |
| *Piabarchus analis* | LBP 8514 | 38382 | Rio Salobra/Cáceres/MT/Brazil | S 15°19'53.5' W 57°11'31.1'' |
| *Piabina argentea* | LBP 3509 | 21306 | Córrego da Hortelã/Botucatu/ SP/Brazil | S 22°56'28.9'' W 48°35'03.2'' |
| *Prionobrama paraguayensis* | LBP 3230 | 19465 | Lagoa marginal/Nobres/ MT/Brazil | S 14°40'32,8'' W 56°13'14,0'' |
| *Prionobrama paraguayensis* | LBP 3230 | 19468 | Lagoa marginal/Nobres/MT/ Brazil | S 14°40'32,8'' W 56°13'14,0'' |
| *Pristella maxillaris* | LBP 2221 | 15637 | Laguna de Castilleros/Caicara del Orinoco/Bolivar/Venezuela | N 07º30’50,9’’ W 66º09’19,8’’ |
| *Probolodus heterostomus* | LBP 6454 | 29141 | Rio Paraíba do Sul/ Guararema/SP/Brazil | S 23°21'38.2'' W 45°59'69.0'' |
| *Psellogrammus kennedyi* | LBP 6578 | 31813 | Lagoa marginal Rio Paraná/Marilena/PR/Brazil | S 22°38'49.4' W 53°04'36.9'' |
| *Rachoviscus crassiceps* | LBP 7146 | 33170 | Riacho sem nome/Guaratuba/ PR/Brazil | S 25°55'27.6'' W 48°36'39.5'' |
| *Roeboexodon guyanensis* | LBP 5315 | 26921 | Igarapé Uiratapuru/Laranjal do Jari/AP/Brazil | S 00°34’03’’ W 52°34’41’’ |
| *Salminus Braziliensis* | LBP 850 | 9025 | Rio Mogi-Guaçu/Pirassununga/ SP/Brazil | S 21°55'37.6'' W 47°22'04.4'' |
| *Salminus franciscanus* | LBP 8090 | 37503 | Rio São Francisco/Três Marias/ MG/Brazil | S 18°11'21.0' W 45°15'10.3'' |
| *Stygichthys typhlops* | LBP 8107 | 37558 | Cacimba fazenda Lajeado/ Jaíba/MG/Brazil | S 15°24'41.7' W 43°45'19.7'' |
| *Thayeria obliqua* | LBP 5743 | 26891 | Lagoa Marginal rio Corrente/ Barra do Garças/MT/Brazil | S 15°19'57.6'' W 52°12'10.4'' |
| *Xenagoniates bondi* | LBP 3074 | 19694 | Rio Orinoco/Caicara del Orinoco/Bolivar/Venezuela | N 07°38'11.6'' W 66°19'04.2'' |
|  |  |  |  |  |
| Acestrorhynchidae |  |  |  |  |
| *Acestrorhynchus falcatus* | LBP 4191 | 23707 | Igarapé Boiboi/Barcelos/ AM/Brazil | S 00°49'43.7'' W 62°49'59.8'' |
| *Acestrorhynchus lacustris* | LBP 2158 | 15173 | Rio Tietê/Botucatu/SP/Brazil | S 27°37' W 48°10' |
| *Acestrorhynchus cf. nasutus* | LBP 7035 | 34110 | Igarapé Ya-Mirim/São Gabriel da Cachoeira/AM/Brazil | N 00°16.259' W 66°38.365' |
| *Acestrorhynchus pantaneiro* | LBP 3755 | 22014 | Rio Negro/Aquidauana/ MS/Brazil | S 19°34'33.7' W 56°14'49.5'' |
|  |  |  |  |  |
| Alestidae |  |  |  |  |
| *Alestes sp.^3^* | LBP 7530 | 35376 | Without procedence |  |
| *Alestopetersius caudalis*^1^ |  |  |  |  |
| *Bathyaethiops breuseghemi*^1^ | AMNH233422 |  |  |  |
| *Brycinus carolinae*^1^ | AMNH233628 |  |  |  |
| *Brycinus longipinnis^3^* | LBP 7529 | 35375 | Without procedence |  |
| *Bryconaethiops sp.* ^1^ |  |  |  |  |
| *Hydrocynus brevis*^1^ | AMNH22644 |  |  |  |
| *Ladigesia roloffi*^1^ | AMNH233394 |  |  |  |
| *Micralestes sp.* | LBP 2342 | 15946 | Aquarium/Brazil |  |
| *Phenacogrammus interruptus* | LBP 2637 | 17293 | Aquarium/Brazil |  |
|  |  |  |  |  |
| Anostomidae |  |  |  |  |
| *Anostomus ternetzi* | LBP 4375 | 24146 | Igarapé Água Azul/Mucajaí/ RR/Brazil | N 02°18'02.0" W 60°55'20.7" |
| *Leporinus fasciatus* | LBP 4459 | 24381 | Igarapé Zalala/Barcelos/ AM/Brazil | S 00°40'03.1'' W 62°58'23.5'' |
| *Schizodon fasciatus* | LBP 3046 | 19130 | Rio Orinoco/Caicara del Orinoco/Bolivar/Venezuela | N 07°38'11.6'' W 66°19'04.2'' |
| *Schizodon fasciatus* | LBP 3994 | 23098 | Lago Morto/São Félix do Araguaia/MT/Brazil | S 11°40'9'' W 50°51'0.30'' |
|  |  |  |  |  |
| Chilodontidae |  |  |  |  |
| *Chilodus punctatus* | LBP 4090 | 23527 | Rio Japiim/Mâncio Lima/AC/ Brazil | S 07°34'28.8' W 72°55'24.9'' |
| *Caenotropus labyrinthicus* | LBP 1828 | 12912 | Rio Araguaia/Aragarças/ GO/Brazil | S 15°53'35.6'' W 52°15'01.0'' |
| *Caenotropus labyrinthicus* | LBP 9216 | 43161 | Rio Apure/Orinoco/Cabruta/ Guárico/Venezuela | N 7°37'24.4" W 66°24'48.0" |
|  |  |  |  |  |
| Citharinidae |  |  |  |  |
| *Citharinus sp.^3^* | LBP 7528 | 35374 | Without procedence |  |
|  |  |  |  |  |
| Crenuchidae |  |  |  |  |
| *Characidium laterale* | LBP 7614 | 36938 | Lagoa Margina rio Cuiabá/ Barão de Melgaço/ MT/Brazil | S 16°11'39.5'' W 55°48'25.1'' |
| *Characidium pterostictum* | LBP 2132 | 21388 | Rio do Peixe/Pedro de Toledo/SP/Brazil | S 24°16'35.5'' W 47°13'33.2'' |
| *Crenuchus spilurus* | LBP 6907 | 33264 | Igarapé Km 50 BR307/São Gabriel da Cachoeira/AM/Brazil | N 00°06.803' W 66°48.744' |
| *Melanocharacidium sp.*^1^ | AMNH233321 |  |  |  |
| *Poecilocharax weitzmani* | LBP 7078 | 40500 | Afluente rio Miuá/São Gabriel da Cachoeira/AM/Brazil | S 00°06.119' W 66°53.756' |
|  |  |  |  |  |
| Ctenoluciidae |  |  |  |  |
| *Boulengerella lateristriga* | LBP 7094 | 34623 | Igarapé margem direita rio Negro/São Gabriel da Cachoeira/AM/Brazil | S 00°08.625' W 67°05.605' |
| *Boulengerella maculata* | LBP 3996 | 23092 | Lago Morto/São Félix do Araguaia/MT/Brazil | S 11°40'9'' W 50°51'0.30'' |
| *Boulengerella maculata* | LBP 4241 | 22733 | Rio Juruá/Cruzeiro do Sul/AC/Brazil | S 07°09'49.6' W 73°43'29.7'' |
| *Ctenolucius hujeta* | LBP 6131 | 29532 | Rio Apon/Machiques de Perijá/Zulia/Venezuela | N 10°09'42.0' W 72°25'58.0'' |
| *Ctenolucius hujeta* | LBP 6131 | 29533 | Rio Apon/Machiques de Perijá/Zulia/Venezuela | N 10°09'42.0' W 72°25'58.0'' |
|  |  |  |  |  |
| Curimatidae |  |  |  |  |
| *Curimatella dorsalis* | LBP3759 | 22034 | Rio Negro/Aquidauana/MS/ Brazil | S 19°34'33.7' W 56°14'49.5'' |
| *Cyphocharax gouldingi* | LBP 1537 | 11889 | Córrego Rola/Aragarças/GO/ Brazil | S 15°53'53.4’' W 52°13'00.6'’ |
| *Cyphocharax magdalenae* | LBP6109 | 29560 | Rio Santa Rosa/Machiques de Perijá/Zulia/Venezuela | N 09°38'53.8' W 72°34'56.4'' |
| *Potamorhina altamazonica* | LBP2571 | 17020 | Lago do Silêncio/Boca do Acre/AM/Brazil | S 08°51'21.5'' W 68°42'22.6'' |
| *Steindachnerina insculpta* | LBP5185 | 26336 | Rio Paraná/Porto Rico/PR/ Brazil | S 22°47’29’’ W 53°20’58’’ |
|  |  |  |  |  |
| Cynodontidae |  |  |  |  |
| *Cynodon gibbus* | LBP 1619 | 11672 | Rio Allipén/Chile | S 39°00'08'’ W 72°26'56'' |
| *Gilbertolus maracaiboensis* | LBP 6107 | 29552 | Rio Santa Rosa/Machiques de Perijá/Zulia/Venezuela | N 09°38'53.8' W 72°34'56.4'' |
| *Hydrolycus scomberoides* | LBP 3031 | 19115 | Rio Orinoco/Caicara del Orinoco/Bolivar/Venezuela | N 07°38'11.6'' W 66°19'04.2'' |
| *Rhaphiodon vulpinus* | LBP 4064 | 22942 | Rio Paraná/Ilha Solteira/SP/ Brazil | S 20°05'07.3' W 50°58'59'' |
| *Roestes ogilviei* | LBP 8157 | 38066 | Lago do Reis/Caracaraí/RR/ Brazil | S 02°00'22.2'' W 61°02'02.9'' |
|  |  |  |  |  |
| Distichodontidae |  |  |  |  |
| *Distichodus sp. ^3^* | LBP 7526 | 35371 | Without procedence |  |
| *Distichodus sp. ^3^* | LBP 7526 | 35372 | Without procedence |  |
| *Hemigrammocharax multifasciatus*^1^ | RUSI63497 |  |  |  |
| *Ichthyborus sp.* ^1^ | AMNH233626 |  |  |  |
| *Neolebias trilineatus*^1^ | AMNH233439 |  |  |  |
| *Xenocharax spilurus*^1^ | AMNH231548 |  |  |  |
|  |  |  |  |  |
| Erythrinidae |  |  |  |  |
| *Erythrinus erythrinus* | LBP 5212 | 26378 | Rio Paraná/Porto Rico/PR/ Brazil | S 22°47’29’’ W 53°20’58’’ |
| *Hoplerythrinus unitaeniatus* | LBP 8025 | 37723 | Riacho sem nome/Nova Mutum/MT/ Brazil | S 13°52'14.7'' W 56°11'30.8'' |
| *Hoplias aimara* | LBP 7837 | 36847 | Lagoa da Boca Franca/ Cocalinho/MT/Brazil | S 13°19' W 50°37' |
| *Hoplias malabaricus* | LBP 5539 | 27219 | Brejo das Ovelhas/Santa Filomena/ PI/ Brazil | S 09°08'04’ W 45°53'48' |
| *Hoplias microlepis* | LBP 2763 | 18503 | Río Llano Sucio/Santa Rita Arriba/Colón/Panamá | N 09°19’26.2'' W 79°46'08.2'' |
|  |  |  |  |  |
| Gasteropelecidae |  |  |  |  |
| *Carnegiella strigata* | LBP 4200 | 23798 | Igarapé Puxirituba/Barcelos/ AM/Brazil | S 00°53'18.6'' W 62°40'36/1'' |
| *Carnegiella marthae* | LBP 4199 | 23793 | Igarapé Puxirituba/Barcelos/ AM/Brazil | S 00°53'18.6'' W 62°40'36/1'' |
| *Gasteropelecus sternicla* | LBP 4070 | 22975 | Rio Japiim/Mâncio Lima/AC/ Brazil | S 07°34'28.8' W 72°55'24.9'' |
| *Thoracocharax stellatus* | LBP 7534 | 35343 | Rio Cuiabá/Cuiabá/MT/Brazil | S 15°39'09.9'' W 56°04'08.6'' |
|  |  |  |  |  |
| Hemiodontidae |  |  |  |  |
| *Anodus orinocensis* | LBP 2210 | 15614 | Laguna de Castilleros/Caicara del Orinoco/Bolivar/Venezuela | N 07º30’50,9’’ W 66º09’19,8’’ |
| *Argonectes robertsi* | LBP 1804 | 13167 | Rio Araguaia/Barra do Garças/MT/Brazil | S 15°32' W 52°12' |
| *Bivibranchia velox* | LBP 5757 | 28123 | Rio Araguaia/Aragarças/GO/ Brazil | S 15°53'31.5'' W 52°15'02.0'' |
| *Hemiodus immaculatus* | LBP1725 | 12849 | Rio Tarumã/Manaus/AM/Brazil | S 02°03'10.0'' W 60°06'31.7'' |
|  |  |  |  |  |
| Hepsetidae |  |  |  |  |
| *Hepsetus odoe^3^* | LBP 7527 | 35373 | Without procedence |  |
|  |  |  |  |  |
| Lebiasinidae |  |  |  |  |
| *Copella nattereri* | LBP 4377 | 24148 | Riacho entorno da Reserva Viruá/Boa Vista/RR/Brazil | N 01°25'25.5'' W 60°59'06.4'' |
| *Copella nattereri* | LBP 536 | 7140 | Igarapé do Leão/Manaus/ AM/Brazil | S 02°53.108’ W 60°02.034’ |
| *Pyrrhulina australis* | LBP 3784 | 22333 | Lagoa Marginal Rio Negro/ Aquidauana/MS/Brazil | S 19°34'17.3' W 56°14'44.8'' |
| *Pyrrhulina cf. zigzag* | LBP 8005 | 37473 | Lagoa permanente/Nova Mutum/MT/Brazil | S 14°08'07.2'' W 56°04'51.5'' |
|  |  |  |  |  |
| Parodontidae |  |  |  |  |
| *Apareiodon affinis* | LBP 4591 | 24665 | Rio Paranapanema/Salto Grande/SP/Brazil | S 22°54'15.4'' W 50°00'03.8'' |
| *Parodon nasus* | LBP 1135 | 5635 | Rio Capivara/Botucatu/ SP/Brazil | S 22°52' W 48°23' |
|  |  |  |  |  |
| Prochilodontidae |  |  |  |  |
| *Prochilodus reticulatus* | LBP 6127 | 29514 | Rio Catatumbo/Catatumbo/ Zulia/ Venezuela | N 09°05'08.3' W 72°13'50.5'' |
| *Semaprochilodus laticeps* | LBP 1383 | 12728 | Caicara del Orinoco/Estado Bolivar/Venezuela | N 7°38'30" W 66°09'00" |
|  |  |  |  |  |
| Serrasalmidae |  |  |  |  |
| *Catoprion mento* | LBP 7556 | 35624 | Lagoa Margina/Barão de Melgaço/MT/Brazil | S 16°11'39.5' W 55°48'25.1'' |
| *Colossoma macropomum* | LBP 5173 | 26648 | Rio Amazonas/Belém/PA/Brazil | S 01°18’20’’ W 48°36’28’’ |
| *Metynnis mola* | LBP 667 | 8050 | Região de Rombado, afluente rio Pirai/Poconé/MT/Brazil | S 16°25,680' W 56°25,143' |
| *Metynnis lippincottianus* | LBP 6282 | 29688 | Córrego Barbacena/Pontal/SP/ Brazil | S 20°56'49.5'' W 48°08'51.9'' |
| *Myloplus rubripinnis* | LBP 2184 | 15570 | Laguna de Castilleros/Caicara del Orinoco/Bolivar/Venezuela | N 07º30’50,9’’ W 66º09’19,8’’ |
| *Mylossoma duriventre* | LBP 1823 | 12921 | Rio Araguaia/Barra do Garças/ MT/Brazil | S 15°32' W 52°12' |
| *Piaractus mesopotamicus* | LBP 4255 | 23803 | CAUNESP/Jaboticabal/SP/ Brazil | Piscicultura |
| *Pygocentrus cariba* | LBP 2229 | 15662 | Afluente do rio Orinoco (Punta Brava)/Caicara del Orinoco /Bolivar/Venezuela | N 07º37’20,7’’ W 66º06’28,8’’ |
| *Serrasalmus maculatus* | LBP 3698 | 21836 | Rio Paraná/Ilha Solteira/SP/ Brazil | S 20°05'07.3' W 50°58'59'' |
| *Serrasalmus spilopleura* | LBP 3499 | 20169 | Rio Tietê/Birigui/SP/Brazil | S 21°06'25.2'' W 50°15'52.7'' |
| *Tometes trilobatus* | LBP 9072 | 42585 | Rio Guamá/Ourém/PA/Brazil | S 01°34'17.0'' W 47°10'10.5'' |
|  |  |  |  |  |
| Cypriniformes |  |  |  |  |
| *Carassius auratus* | LBP 9215 | 43160 | Aquarium/Brazil |  |
| *Gyrinocheilus sp*.^1^ | AMNH233433 |  |  |  |

1- Calcagnotto et al. [32]; 2- Javonillo et al. [33]; 3 – Guillermo Ortí personal collection.
