# Supplementary material for: Phylogenetic relationships within the speciose family Characidae (Teleostei: Ostariophysi: Characiformes) based on multilocus analysis and extensive ingroup sampling
Source: BMC Evol Biol. 2011 Sep 26;11:275. doi: 10.1186/1471-2148-11-275 (PMC3190395; doi:10.1186/1471-2148-11-275)
Supplement: Additional file 2 — Sequences of primers used in present study. [file 1471-2148-11-275-S2.DOC]

Additional File 2 - Sequences of primers used in present study.

| **Gene** | **Primer name** | **Primer sequence (5’-3’)** | **Source** |
| --- | --- | --- | --- |
| 16S | 16Sa-L | ACGCCTGTTTATCAAAAACAT | [1] |
|  | 16Sb-H | CCGGTCTGAACTCAGATCACGT | [1] |
|  |  |  |  |
| Cytb | L14841 | AAATCAAAGCATAACACTGAAGATG | [2] |
|  | H15915 | CCAATTTGCATGGATGTCTTCTCGG | [3] |
|  | LNF | GACTTGAAAAACCAYCGTTGT | Present study |
|  | H08R2 | GCTTTGGGAGTTAGDGGTGGGAGTTAGAATC | Present study |
|  |  |  |  |
| Myh6 | F329 | CCGCMTGGATGATCTACAC | [4] |
| 1stPCR | A3R1 | ATTCTCACCACCATCCAGTTGAA | [4] |
| Myh6 | A3F2 | GGAGAATCARTCKGTGCTCATCA | [4] |
| 2ndPCR | A3R2 | CTCACCACCATCCAGTTGAACAT | [4] |
|  | R1242 | ACAGGATTGAGATGCTGTCCA | [4] |
|  | Myh6COF1 | GACTGTTAACACCAAGAGAGT | Present study |
|  | Myh6COF2 | GTTATCCAGTATTTTGCAAGTATTGC | Present study |
|  | Myh6COR1 | TTGAACATCTTCTCATACAC | Present study |
|  | Myh6COR2 | TTCTCATACACTGACTTAGCCAGTGC | Present study |
|  |  |  |  |
| RAG1 | 2510F | TGGCCATCCGGGTMAACAC | [5] |
| 1stPCR | 4090R | CTGAGTCCTTGTGAGCTTCCATRAAYTT | [5] |
| RAG1 | 2535F | AGCCAGTACCATAAGATGTA | [5] |
| 2ndPCR | 4078R | TGAGCCTCCATGAACTTCTGAAGRTAYTT | [5] |
|  | Rag1CF1 | ACCCTCCGTACTGCTGAGAA | Present study |
|  | Rag1CF2 | TACCGCTGAGAAGGAGCTTC | Present study |
|  | Rag1CF3 | GAGAAGGAGCTTCTCCCAGG | Present study |
|  | Rag1CF4 | GCTTCCATCAGTTTGAGTGG | Present study |
|  | Rag1CF5 | CAGCTCTTGGAACATAGGCATCA | Present study |
|  | Rag1CR1 | CGTCGGAAGAGCTTGTTGCC | Present study |
|  | Rag1CR2 | TGTTGCCAGACTCATTGCCCTC | Present study |
|  | Rag1CR3 | CCCTCGCTGGCCCAGGCACC | Present study |
|  | Rag1CR4 | ATCTCGTTCCACAATCTCAGGC | Present study |
|  | Rag1CR5 | CATGGGCCAGTGTCTTGTGGAGGT | Present study |
|  |  |  |  |
| RAG2 | 164F | AGCTCAAGCTGCGYGCCAT | Present study |
| 1stPCR | RAG2-R6 | TGRTCCARGCAGAAGTACTTG | [6] |
| RAG2 | 176R | GYGCCATCTCATTCTCCAACA | Present study |
| 2ndPCR | Rag2Ri | AGAACAAAAGATCATTGCTGGTCGGG | Present study |

References

1. Palumbi SR: **Nucleic acids II: the polymerase chain reaction**. In *Molecular Systematics*. Edited by Hillis D, Moritz C, Mable B. Massachusetts: Sinauer Associates Inc.; 1996:205-247.

2. Kocher TD, Thomas WK, Meyer A, Edwards SV, Pääbo S, Villablanca FX, Wilson A: **Dynamics of mitochondrial DNA evolution in animals: ampliWcation and sequencing with conserved primers**. *Proc Natl Acad Sci* 1989, **86**:6196-6200.

3. Irwing DM, Kocher TD, Wilson AC: **Evolution of the cytochrome *b* gene of mammals**. *J Mol Evol* 1991, **32**:128-144.

4. Li C, Ortí G, Zhang G, Lu G: **A practical approach to phylogenomics: The phylogeny of ray-finned fish (Actinopterygii) as a case study**. *BMC Evol Biol* 2007, **7**:44.

5. Li C, Ortí G: **Molecular phylogeny of Clupeiformes (Actinopterygii) inferred from nuclear and mitochondrial DNA sequences**. *Mol Phylogenet Evol* 2007, **44**:386-398.

6. Lovejoy NR, Collette BB: **Phylogenetic relationships of new world needlefishes (Teleostei: Belonidae) and the biogeography of transitions between marine and freshwater habitats**. *Copeia* 2001, 1: 324–338.
