# Supplementary material for: Phylogenetic relationships within the speciose family Characidae (Teleostei: Ostariophysi: Characiformes) based on multilocus analysis and extensive ingroup sampling
Source: BMC Evol Biol. 2011 Sep 26;11:275. doi: 10.1186/1471-2148-11-275 (PMC3190395; doi:10.1186/1471-2148-11-275)
Supplement: Additional file 3 — Species analyzed, collection number, specimen number, and GenBank accession numbers. [file 1471-2148-11-275-S3.DOC]

Additional File 3 - Species analyzed, collection number, specimen number, and GenBank accession numbers.

| **Group/species** | **Voucher** | **Specimen** | **16S** | **CytB** | **Myh6** | **Rag1** | **Rag2** |
| --- | --- | --- | --- | --- | --- | --- | --- |
| Characidae/Agoniatinae |  |  |  |  |  |  |  |
| *Agoniates anchovia* | LBP 6740 | 33471 | HQ171378 | HQ289665 | - | - | HQ289472 |
| *Agoniates halecinus* | LBP 5503 | 26594 | HQ171342 | HQ289631 | HQ289051 | HQ289280 | HQ289437 |
|  |  |  |  |  |  |  |  |
| Characidae/Aphyocharacinae |  |  |  |  |  |  |  |
| *Aphyocharax alburnus* | LBP 1587 | 11774 | HQ171242 | HQ289533 | HQ288952 | HQ289149 | - |
| *Aphyocharax anisitsi* | LBP 3764 | 22190 | HQ171292 | HQ289581 | HQ289002 | HQ289196 | HQ289389 |
| *Aphyocharax pusillus* | LBP 4046 | 22920 | HQ171301 | HQ289590 | HQ289011 | HQ289203 | HQ289397 |
|  |  |  |  |  |  |  |  |
| Characidae/Bryconinae |  |  |  |  |  |  |  |
| *Brycon amazonicus* | LBP 2187 | 15565 | HQ171251 | HQ289542 | HQ288961 | HQ289158 | HQ289349 |
| *Brycon insignis* | LBP 2369 | 16075 | HQ171260 | HQ289551 | HQ288970 | HQ289167 | HQ289358 |
| *Henochilus wheatlandii* | LBP 1221 | 25846 | HQ171335 | HQ289624 | HQ289044 | HQ289237 | HQ289431 |
|  |  |  |  |  |  |  |  |
| Characidae/Characinae |  |  |  |  |  |  |  |
| *Acestrocephalus sardina* | LBP 6876 | 33172 | HQ171373 | HQ289660 | - | HQ289274 | HQ289467 |
| *Charax leticiae* | LBP 1480 | 12700 | HQ171244 | HQ289535 | HQ288954 | HQ289151 | HQ289342 |
| *Cynopotamus kincaidi* | LBP 3225 | 19449 | HQ171271 | HQ289560 | HQ288981 | HQ289178 | HQ289368 |
| *Cynopotamus venezuelae* | LBP 6132 | 29515 | HQ171359 | HQ289648 | HQ289068 | HQ289261 | HQ289454 |
| *Galeocharax knerii* | LBP 3496 | 20164 | HQ171280 | HQ289569 | HQ288990 | - | HQ289377 |
| *Gnathocharax steindachneri* | LBP 4496 | 24494 | HQ171325 | - | HQ289034 | - | HQ289421 |
| *Heterocharax macrolepis* | LBP 4494 | 24485 | HQ171323 | HQ289612 | HQ289032 | HQ289225 | HQ289419 |
| *Hoplocharax goethei* | LBP 4495 | 24489 | HQ171324 | HQ289613 | - | HQ289226 | - |
| *Phenacogaster calverti* | LBP 5582 | 27299 | HQ171347 | HQ289636 | HQ289056 | HQ289249 | HQ289442 |
| *Roeboides guatemalensis* | LBP 2755 | 18529 | HQ171267 | - | - | - | HQ289364 |
|  |  |  |  |  |  |  |  |
| Characidae/Cheirodontinae |  |  |  |  |  |  |  |
| *Aphyocheirodon hemigrammus* | LBP 8306 | 40025 | HQ171413 | HQ289700 | HQ289121 | HQ289314 | - |
| *Cheirodon killiani* | LBP 3115 | 19803 | HQ171275 | HQ289564 | - | - | HQ289372 |
| *Cheirodon ibicuhiensis* | LBP 4777 | 25598 | HQ171334 | HQ289623 | HQ289043 | HQ289236 | HQ289430 |
| *Compsura heterura* | LBP 4733 | 24984 | HQ171332 | HQ289621 | HQ289041 | HQ289234 | HQ289428 |
| *Heterocheirodon yatai* | LBP 4872 | 24954 | HQ171330 | HQ289619 | HQ289039 | HQ289232 | HQ289426 |
| *Kolpotocheirodon theloura* | LBP 5033 | 25982 | HQ171336 | HQ289625 | HQ289045 | HQ289238 | HQ289432 |
| *Macropsobrycon uruguayanae* | LBP 6039 | 29061 | HQ171355 | HQ289644 | HQ289064 | HQ289257 | HQ289450 |
| *Nanocheirodon insignis* | LBP 6104 | 27476 | HQ171349 | HQ289638 | HQ289058 | HQ289251 | HQ289444 |
| *Odontostilbe sp.* | LBP 4650 | 22626 | HQ171296 | HQ289585 | HQ289006 | HQ289200 | HQ289393 |
| *Odontostilbe fugitiva* | LBP 4052 | 22932 | HQ171302 | HQ289591 | HQ289012 | HQ289204 | HQ289398 |
| *Prodontocharax melanotus* | AMNH | 233264 | HQ 171239 | HQ289530 | HQ288949 | HQ289146 | HQ289337 |
| *Pseudocheirodon arnoldi* | STRI | 5 | HQ171430 | HQ289715 | HQ289138 | HQ289329 | HQ289522 |
| *Saccoderma melanostigma* | LBP 6103 | 27475 | HQ171348 | HQ289637 | HQ289057 | HQ289250 | HQ289443 |
| *Serrapinnus calliurus* | LBP 3731 | 22121 | HQ171291 | HQ289580 | HQ289001 | HQ289195 | HQ289388 |
| *Serrapinnus heterodon* | LBP 9039 | 37551 | HQ171403 | HQ289690 | HQ289111 | HQ289304 | HQ289496 |
| *Serrapinnus piaba* | LBP 8972 | 41813 | HQ171416 | HQ289703 | HQ289124 | HQ289317 | HQ289509 |
| *Spintherobolus ankoseion* | LBP 4725 | 24957 | HQ171331 | HQ289620 | HQ289040 | HQ289294 | HQ289427 |
| *Spintherobolus broccae* | LBP 3916 | 22558 | HQ171294 | HQ289583 | HQ289004 | HQ289233 | HQ289391 |
| *Spintherobolus leptoura* | LBP 7544 | 36098 | HQ171393 | HQ289680 | HQ289101 | HQ289198 | HQ289486 |
| *Gen. & sp. nov.* | LBP 5699 | 27603 | HQ171350 | HQ289639 | HQ289059 | HQ289252 | HQ289445 |
|  |  |  |  |  |  |  |  |
| Characidae/Clupeacharacinae |  |  |  |  |  |  |  |
| *Clupeacharax anchoveoides* | LBP 5046 | 26012 | HQ171337 | HQ289626 | HQ289046 | HQ289239 | HQ289433 |
|  |  |  |  |  |  |  |  |
| Characidae/Glandulocaudinae |  |  |  |  |  |  |  |
| *Glandulocauda melanogenys* | LBP 4507 | 24538 | HQ171320 | HQ289609 | HQ289029 | HQ289222 | HQ289416 |
| *Lophiobrycon weitzmani* | LBP 1225 | 38090 | HQ171411 | HQ289698 | HQ289119 | HQ289312 | HQ289504 |
| *Mimagoniates inequalis* | LBP 3383 | 21274 | HQ171282 | HQ289571 | HQ288992 | HQ289186 | HQ289379 |
| *Mimagoniates microlepis* | LBP 1225 | 11077 | HQ171240 | HQ289531 | HQ288950 | HQ289147 | HQ289338 |
|  |  |  |  |  |  |  |  |
| Characidae/Iguanodectinae |  |  |  |  |  |  |  |
| *Iguanodectes geisleri* | LBP 4266 | 23840 | HQ171316 | HQ289605 | - | - | HQ289412 |
| *Piabucus melanostomus* | LBP 5109 | 26150 | HQ171338 | HQ289627 | HQ289047 | - | - |
|  |  |  |  |  |  |  |  |
| Characidae/Rhoadsiinae |  |  |  |  |  |  |  |
| *Carlana eigenmanni* | LBP 3300 | 19864 | HQ171276 | HQ289565 | HQ288986 | HQ289181 | - |
| *Carlana eigenmanni* | LBP 3301 | 19865 | HQ171277 | HQ289566 | HQ288987 | HQ289182 | - |
|  |  |  |  |  |  |  |  |
| Characidae/Stethaprioninae |  |  |  |  |  |  |  |
| *Brachychalcinus copei* | LBP 192 | 8853 | HQ171435 | HQ289720 | HQ289143 | HQ289334 | HQ289527 |
| *Orthospinus franciscensis* | LBP 8105 | 37555 | HQ171404 | - | HQ289112 | HQ289305 | HQ289497 |
| *Poptella paraguayensis* | LBP 3732 | 21986 | HQ171286 | HQ289575 | HQ288996 | HQ289190 | - |
| *Stethaprion crenatum* | LBP 4078 | 22994 | HQ171305 | HQ289594 | HQ289015 | HQ289207 | HQ289401 |
|  |  |  |  |  |  |  |  |
| Characidae/Stevardiinae |  |  |  |  |  |  |  |
| *Corynopoma riisei*^2^ |  |  | FJ749009 | - | - | - | FJ749095 |
| *Gephyrocharax atracaudatus* | LBP 2753 | 18519 | HQ171265 | HQ289556 | HQ288975 | HQ289172 | HQ289362 |
| *Planaltina britskii* | LBP 2598 | 17243 | HQ171262 | HQ289553 | HQ288972 | HQ289169 | - |
| *Pseudocorynopoma heterandria* | LBP 2862 | 18570 | HQ171268 | - | HQ288978 | HQ289175 | HQ289365 |
| *Tyttocharax madeirae* | LBP 5145 | 33166 | HQ171368 | HQ289655 | HQ289076 | HQ289269 | HQ289462 |
| *Xenurobrycon pteropus* | LBP 9054 | 42218 | HQ171423 | HQ289709 | HQ289131 | HQ289324 | HQ289516 |
|  |  |  |  |  |  |  |  |
| Characidae/Tetragonopterinae |  |  |  |  |  |  |  |
| *Tetragonopterus argenteus* | LBP 3758 | 22029 | HQ171289 | HQ289578 | HQ288999 | HQ289193 | HQ289386 |
| *Tetragonopterus chalceus* | LBP 8268 | 37556 | HQ171405 | HQ289692 | HQ289113 | HQ289306 | HQ289498 |
|  |  |  |  |  |  |  |  |
| Characidae/Triportheinae |  |  |  |  |  |  |  |
| *Lignobrycon myersi* | LBP 8094 | 37519 | HQ171402 | HQ289689 | HQ289110 | HQ289303 | - |
| *Triportheus nematurus* | LBP 39 | 3503 | HQ171383 | HQ289670 | HQ289091 | HQ289284 | HQ289476 |
| *Triportheus orinocensis* | LBP 2663 | 15580 | HQ171253 | HQ289544 | HQ288963 | HQ289160 | HQ289351 |
|  |  |  |  |  |  |  |  |
| Characidae *incertae sedis* |  |  |  |  |  |  |  |
| *Aphyocharacidium bolivianum* | LBP 9055 | 42219 | HQ171424 | HQ289710 | HQ289132 | HQ289325 | HQ289517 |
| *Aphyodite grammica* | LBP 9050 | 42214 | HQ171421 | HQ289707 | HQ289129 | HQ289322 | HQ289514 |
| *Astyanacinus moorii* | LBP 5783 | 28195 | HQ171352 | HQ289641 | HQ289061 | HQ289254 | HQ289447 |
| *Astyanax aeneus* | LBP 8938 | 42019 | HQ171418 | - | HQ289126 | HQ289319 | HQ289511 |
| *Astyanax jordani* | LBP 4527 | 24599 | HQ171327 | HQ289616 | HQ289036 | HQ289229 | HQ289423 |
| *Astyanax mexicanus* | LBP 8937 | 42016 | HQ171417 | - | HQ289125 | HQ289318 | HQ289510 |
| *Bario steindachneri* | LBP 4389 | 24187 | HQ171319 | HQ289608 | HQ289028 | HQ289221 | HQ289415 |
| *Bramocharax baileyi* | LBP 8940 | 42025 | HQ171420 | HQ289706 | HQ289128 | HQ289321 | HQ289513 |
| *Bramocharax caballeroi* | LBP 8939 | 42022 | HQ171419 | HQ289705 | HQ289127 | HQ289320 | HQ289512 |
| *Bryconadenos tanaothoros*^2^ | MCP 40399 |  | FJ748980 | - | - | - | FJ749089 |
| *Bryconamericus emperador* | LBP 2754 | 18528 | HQ171266 | HQ289557 | HQ288976 | HQ289173 | HQ289363 |
| *Bryconamericus exodon* | LBP 7123 | 34200 | HQ171380 | HQ289667 | HQ289088 | HQ289281 | HQ289474 |
| *Bryconella pallidifrons* | LBP 4646 | 24696 | HQ171329 | HQ289618 | HQ289038 | HQ289231 | HQ289425 |
| *Bryconops affinis* | LBP 262 | 4168 | HQ171415 | HQ289702 | HQ289123 | HQ289316 | HQ289508 |
| *Ceratobranchia cf. delotaenia* | LBP 3257 | 20042 | HQ171278 | HQ289567 | HQ288988 | HQ289183 | HQ289375 |
| *Chalceus epakros* | LBP 5443 | 26504 | HQ171341 | HQ289630 | - | - | HQ289436 |
| *Chalceus erythrurus* | LBP 4211 | 22727 | HQ171297 | HQ289586 | HQ289007 | HQ289201 | HQ289394 |
| *Coptobrycon bilineatus* | LBP 3809 | 33169 | HQ171370 | HQ289657 | HQ289078 | HQ289271 | HQ289464 |
| *Creagrutus peruanus* | LBP 3267 | 20057 | HQ171279 | HQ289568 | HQ288989 | HQ289184 | HQ289376 |
| *Ctenobrycon hauxwellianus* | LBP 4095 | 23538 | HQ171310 | HQ289599 | HQ289020 | HQ289212 | HQ289406 |
| *Cyanocharax alburnus* | LBP 4746 | 25516 | HQ171333 | HQ289622 | HQ289042 | HQ289235 | HQ289429 |
| *Deuterodon iguape* | LBP 6827 | 33065 | HQ171366 | HQ289653 | HQ289074 | HQ289267 | HQ289460 |
| *Engraulisoma taeniatum* | LBP 4038 | 22896 | HQ171299 | HQ289588 | HQ289009 | - | - |
| *Engraulisoma taeniatum* | LBP 4038 | 22897 | HQ171300 | HQ289589 | HQ289010 | - | - |
| *Exodon paradoxus* | LBP 4006 | 23040 | HQ171306 | HQ289595 | HQ289016 | HQ289208 | HQ289402 |
| *Gymnocorymbus ternetzi* | LBP 3737 | 21989 | HQ171287 | HQ289576 | HQ288997 | HQ289191 | HQ289384 |
| *Hasemania sp.* | LBP5967 | 28455 | HQ171354 | HQ289643 | HQ289063 | HQ289256 | HQ289449 |
| *Hemibrycon taeniurus* | LBP 6847 | 33168 | HQ171369 | HQ289656 | HQ289077 | HQ289270 | HQ289463 |
| *Hemigrammus marginatus* | LBP 6292 | 29419 | HQ171357 | HQ289646 | HQ289066 | HQ289259 | HQ289452 |
| *Hemigrammus ulreyi* | LBP 7604 | 36267 | HQ171394 | HQ289681 | HQ289102 | HQ289295 | HQ289470 |
| *Hollandichthys multifasciatus* | LBP 698 | 8791 | HQ171434 | - | HQ289142 | HQ289333 | HQ289526 |
| *Hyphessobrycon eques* | LBP 7615 | 36278 | HQ171395 | HQ289682 | HQ289103 | HQ289296 | HQ289488 |
| *Hyphessobrycon megalopterus* | LBP 7613 | 36932 | HQ171397 | HQ289684 | HQ289105 | HQ289298 | HQ289490 |
| *Hyphessobrycon reticulatus* | LBP 1049 | 8939 | HQ171436 | HQ289721 | HQ289144 | HQ289335 | HQ289487 |
| *Hypobrycon maromba* | LBP 6750 | 33174 | HQ171375 | HQ289662 | HQ289083 | HQ289276 | HQ289469 |
| *Inpaichthys kerri* | LBP 4526 | 24597 | HQ171326 | HQ289615 | HQ289035 | HQ289228 | HQ289422 |
| *Jupiaba anteroides* | LBP 7067 | 34380 | HQ171381 | HQ289668 | HQ289089 | HQ289282 | HQ289475 |
| *Jupiaba cf. acanthogaster* | LBP 7935 | 37269 | HQ171399 | HQ289686 | HQ289107 | HQ289300 | HQ289492 |
| *Knodus meridae* | LBP 7569 | 15818 | HQ171257 | HQ289548 | HQ288967 | HQ289164 | HQ289355 |
| *Leptagoniates steindachneri* | LBP 4137 | 23661 | HQ171311 | HQ289600 | HQ289021 | HQ289213 | HQ289407 |
| *Markiana nigripinnis* | LBP 663 | 8038 | HQ171432 | - | HQ289140 | HQ289331 | HQ289524 |
| *Microschemobrycon casiquiare* | LBP 8161 | 38058 | HQ171409 | HQ289696 | HQ289117 | HQ289310 | HQ289502 |
| *Moenkhausia xinguensis* | LBP 6101 | 28443 | HQ171353 | HQ289642 | HQ289062 | HQ289255 | HQ289448 |
| *Myxiops aphos* | LBP 7184 | 33173 | HQ171374 | HQ289661 | HQ289082 | HQ289275 | - |
| *Nematobrycon palmeri* | LBP 6124 | 33165 | HQ171367 | HQ289654 | HQ289075 | HQ289268 | HQ289461 |
| *Nematocharax venustus* | LBP 8106 | 37557 | HQ171406 | HQ289693 | HQ289114 | HQ289307 | HQ289499 |
| *Odontostoechus lethostigmus* | LBP 6752 | 33171 | HQ171372 | HQ289659 | HQ289080 | HQ289273 | HQ289466 |
| *Oligosarcus paranensis* | LBP 3926 | 22582 | HQ171295 | HQ289584 | HQ289005 | HQ289199 | HQ289392 |
| *Oligosarcus hepsetus* | LBP 2377 | 16055 | HQ171259 | HQ289550 | - | - | HQ289357 |
| *Paracheirodon axelrodi* | LBP 4472 | 24425 | HQ171322 | HQ289611 | HQ289031 | HQ289224 | HQ289418 |
| *Paragoniates alburnus* | LBP 9208 | 43156 | HQ171426 | HQ289712 | HQ289134 | HQ289326 | HQ289519 |
| *Parecbasis cyclolepis* | LBP 9053 | 42217 | HQ171422 | HQ289708 | HQ289130 | HQ289323 | HQ289515 |
| *Phenagoniates macrolepis* | LBP 6105 | 35623 | HQ171391 | HQ289678 | HQ289099 | HQ289292 | HQ289484 |
| *Piabarchus analis* | LBP 8514 | 38382 | HQ171412 | HQ289699 | HQ289120 | HQ289313 | HQ289505 |
| *Piabina argentea* | LBP 3509 | 21306 | HQ171283 | HQ289572 | HQ288993 | HQ289187 | HQ289380 |
| *Prionobrama paraguayensis* | LBP 3230 | 19465 | HQ171272 | - | HQ288982 | - | HQ289369 |
| *Prionobrama paraguayensis* | LBP 3230 | 19468 | HQ171273 | - | HQ288983 | HQ289179 | HQ289370 |
| *Pristella maxillaris* | LBP 2221 | 15637 | HQ171255 | HQ289546 | HQ288965 | HQ289162 | HQ289353 |
| *Probolodus heterostomus* | LBP 6454 | 29141 | HQ171356 | HQ289645 | HQ289065 | HQ289258 | HQ289451 |
| *Psellogrammus kennedyi* | LBP 6578 | 31813 | HQ171365 | HQ289652 | HQ289073 | HQ289266 | HQ289459 |
| *Rachoviscus crassiceps* | LBP 7146 | 33170 | HQ171371 | HQ289658 | HQ289079 | HQ289272 | HQ289465 |
| *Roeboexodon guyanensis* | LBP 5315 | 26921 | HQ171345 | HQ289634 | HQ289054 | HQ289247 | HQ289440 |
| *Salminus brasiliensis* | LBP 850 | 9025 | HQ171437 | HQ289722 | HQ289145 | HQ289336 | HQ289528 |
| *Salminus franciscanus* | LBP 8090 | 37503 | HQ171401 | HQ289688 | HQ289109 | HQ289302 | HQ289494 |
| *Stygichthys typhlops* | LBP 8107 | 37558 | HQ171407 | HQ289694 | HQ289115 | HQ289308 | HQ289500 |
| *Thayeria obliqua* | LBP 5743 | 26891 | HQ171344 | HQ289633 | HQ289053 | HQ289246 | HQ289439 |
| *Xenagoniates bondi* | LBP 3074 | 19694 | HQ171274 | HQ289563 | HQ288984 | - | HQ289371 |
| *Gen. & sp. nov.* | LBP 7243 | 33196 | HQ171376 | HQ289663 | - | - | HQ289470 |
|  |  |  |  |  |  |  |  |
| Acestrorhynchidae |  |  |  |  |  |  |  |
| *Acestrorhynchus falcatus* | LBP 4191 | 23707 | HQ171312 | HQ289601 | HQ289022 | HQ289214 | HQ289408 |
| *Acestrorhynchus lacustris* | LBP 2158 | 15173 | HQ171250 | HQ289541 | HQ288960 | HQ289157 | HQ289348 |
| *Acestrorhynchus cf. nasutus* | LBP 7035 | 34110 | HQ171379 | HQ289666 | HQ289087 | HQ289280 | - |
| *Acestrorhynchus pantaneiro* | LBP 3755 | 22014 | HQ171288 | HQ289577 | HQ288998 | HQ289192 | HQ289385 |
|  |  |  |  |  |  |  |  |
| Alestidae |  |  |  |  |  |  |  |
| *Alestes sp.* | LBP 7530 | 35376 | HQ171390 | HQ289677 | HQ289098 | HQ289291 | HQ289483 |
| *Alestopetersius caudalis*^1^ |  |  | AY788019 | AY791401 | - | - | AY804078 |
| *Bathyaethiops breuseghemi*^1^ | AMNH 233422 |  | AY788068 | AY791430 | - | - | AY804113 |
| *Brycinus carolinae*^1^ | AMNH 233628 |  | AY787960 | AY791357 | - | - | - |
| *Brycinus longipinnis* | LBP 7529 | 35375 | HQ171389 | HQ289676 | HQ289097 | HQ289290 | HQ289482 |
| *Bryconaethiops sp.* ^1^ |  |  | AY787983 | AY791374 | - | - | AY804047 |
| *Hydrocynus brevis*^1^ | AMNH 22644 |  | AY788018 | AY791400 | - | - | AY804077 |
| *Ladigesia roloffi*^1^ | AMNH 233394 |  | AY788046 | AY791417 | - | - | AY804097 |
| *Micralestes sp.* | LBP 2342 | 15946 | HQ171258 | HQ289549 | HQ288968 | HQ289165 | HQ289356 |
| *Phenacogrammus interruptus* | LBP 2637 | 17293 | HQ171263 | HQ289554 | HQ288973 | HQ289170 | HQ289361 |
|  |  |  |  |  |  |  |  |
| Anostomidae |  |  |  |  |  |  |  |
| *Anostomus ternetzi* | LBP 4375 | 24146 | HQ171317 | HQ289606 | HQ289026 | HQ289219 | HQ289413 |
| *Leporinus fasciatus* | LBP 4459 | 24381 | HQ171321 | HQ289610 | HQ289030 | HQ289223 | HQ289417 |
| *Schizodon fasciatus* | LBP 3046 | 19130 | HQ171270 | HQ289559 | HQ288980 | HQ289177 | HQ289367 |
| *Schizodon fasciatus* | LBP 3994 | 23098 | HQ171308 | HQ289597 | HQ289018 | HQ289210 | HQ289404 |
|  |  |  |  |  |  |  |  |
| Chilodontidae |  |  |  |  |  |  |  |
| *Chilodus punctatus* | LBP 4090 | 23527 | HQ171309 | HQ289598 | - | - | - |
| *Caenotropus labyrinthicus* | LBP 1828 | 12912 | HQ171247 | HQ289538 | - | - | HQ289345 |
| *Caenotropus labyrinthicus* | LBP 9216 | 43161 | HQ171428 | - | HQ289136 | HQ289327 | - |
|  |  |  |  |  |  |  |  |
| Citharinidae |  |  |  |  |  |  |  |
| *Citharinus sp.* | LBP 7528 | 35374 | HQ171388 | HQ289675 | - | - | HQ289481 |
|  |  |  |  |  |  |  |  |
| Crenuchidae |  |  |  |  |  |  |  |
| *Characidium laterale* | LBP 7614 | 36938 | HQ171398 | HQ289685 | HQ289106 | HQ289299 | HQ289491 |
| *Characidium pterostictum* | LBP 2132 | 21388 | HQ171284 | HQ289573 | HQ288994 | HQ289188 | HQ289381 |
| *Crenuchus spilurus* | LBP 6907 | 33264 | HQ171377 | HQ289664 | HQ289085 | HQ289278 | HQ289471 |
| *Melanocharacidium sp.*^1^ | AMNH 233321 |  | AY788083 | AY791439 | - | - | AY804126 |
| *Poecilocharax weitzmani* | LBP 7078 | 40500 | HQ171414 | HQ289701 | HQ289122 | HQ289315 | HQ289507 |
|  |  |  |  |  |  |  |  |
| Ctenoluciidae |  |  |  |  |  |  |  |
| *Boulengerella lateristriga* | LBP 7094 | 34623 | HQ171382 | HQ289669 | - | - | - |
| *Boulengerella maculata* | LBP 3996 | 23092 | HQ171307 | HQ289596 | - | - | - |
| *Boulengerella maculata* | LBP 4241 | 22733 | HQ171298 | HQ289587 | - | - | - |
| *Ctenolucius hujeta* | LBP 6131 | 29532 | HQ171360 |  | - | - | - |
| *Ctenolucius hujeta* | LBP 6131 | 29533 | HQ171361 | - | - | - | - |
|  |  |  |  |  |  |  |  |
| Curimatidae |  |  |  |  |  |  |  |
| *Curimatella dorsalis* | LBP3759 | 22034 | HQ171290 | HQ289579 | HQ289000 | HQ289194 | HQ289387 |
| *Cyphocharax gouldingi* | LBP 1537 | 11889 | HQ171243 | HQ289534 | HQ288953 | HQ289150 | HQ289342 |
| *Cyphocharax magdalenae* | LBP6109 | 29560 | HQ171363 | HQ289650 | HQ289071 | HQ289264 | - |
| *Potamorhina altamazonica* | LBP2571 | 17020 | HQ171261 | HQ289552 | HQ288971 | HQ289168 | HQ289359 |
| *Steindachnerina insculpta* | LBP5185 | 26336 | HQ171339 | HQ289628 | HQ289048 | HQ289241 | HQ289435 |
|  |  |  |  |  |  |  |  |
| Cynodontidae |  |  |  |  |  |  |  |
| *Cynodon gibbus* | LBP 1619 | 11672 | HQ171241 | HQ289532 | HQ288951 | HQ289148 | HQ289339 |
| *Gilbertolus maracaiboensis* | LBP 6107 | 29552 | HQ171362 | HQ289649 | HQ289070 | HQ289263 | HQ289456 |
| *Hydrolycus scomberoides* | LBP 3031 | 19115 | HQ171269 | HQ289558 | HQ288979 | HQ289176 | HQ289366 |
| *Rhaphiodon vulpinus* | LBP 4064 | 22942 | HQ171303 | HQ289592 | HQ289013 | HQ289205 | - |
| *Roestes ogilviei* | LBP 8157 | 38066 | HQ171410 | HQ289697 | HQ289118 | HQ289311 | HQ289503 |
|  |  |  |  |  |  |  |  |
| Distichodontidae |  |  |  |  |  |  |  |
| *Distichodus sp.* | LBP 7526 | 35371 | HQ171385 | HQ289672 | HQ289093 | HQ289286 | HQ289478 |
| *Distichodus sp.* | LBP 7526 | 35372 | HQ171386 | HQ289673 | HQ289094 | HQ289287 | HQ289479 |
| *Hemigrammocharax multifasciatus*^1^ | RUSI63497 |  | AY788029 | AY791407 | - | - | AY804085 |
| *Ichthyborus sp.* ^1^ | AMNH 233626 |  | AY788038 | AY791412 | - | - | AY804092 |
| *Neolebias trilineatus*^1^ | AMNH 233439 |  | AY788063 | AY791425 | - | - | AY804108 |
| *Xenocharax spilurus*^1^ | AMNH 231548 |  | AY788085 | AY791441 | - | - | - |
|  |  |  |  |  |  |  |  |
| Erythrinidae |  |  |  |  |  |  |  |
| *Erythrinus erythrinus* | LBP 5212 | 26378 | HQ171340 | HQ289629 | HQ289049 | HQ289242 | - |
| *Hoplerythrinus unitaeniatus* | LBP 8025 | 37723 | HQ171408 | - | HQ289116 | HQ289309 | - |
| *Hoplias aimara* | LBP 7837 | 36847 | HQ171396 | HQ289683 | HQ289104 | HQ289248 | - |
| *Hoplias malabaricus* | LBP 5539 | 27219 | HQ171346 | HQ289635 | HQ289055 | HQ289297 | - |
| *Hoplias microlepis* | LBP 2763 | 18503 | HQ171264 | HQ289555 | HQ288974 | HQ289171 | - |
|  |  |  |  |  |  |  |  |
| Gasteropelecidae |  |  |  |  |  |  |  |
| *Carnegiella strigata* | LBP 4200 | 23798 | HQ171314 | HQ289603 | - | HQ289215 | HQ289410 |
| *Carnegiella marthae* | LBP 4199 | 23793 | HQ171313 | HQ289602 | HQ289023 | HQ289216 | HQ289409 |
| *Gasteropelecus sternicla* | LBP 4070 | 22975 | HQ171304 | - | HQ289014 | HQ289206 | HQ289400 |
| *Thoracocharax stellatus* | LBP 7534 | 35343 | HQ171384 | HQ289671 | HQ289092 | HQ289285 | - |
|  |  |  |  |  |  |  |  |
| Hemiodontidae |  |  |  |  |  |  |  |
| *Anodus orinocensis* | LBP 2210 | 15614 | HQ171254 | HQ289545 | HQ288964 | HQ289161 | HQ289352 |
| *Argonectes robertsi* | LBP 1804 | 13167 | HQ171249 | HQ289540 | HQ288959 | HQ289156 | HQ289347 |
| *Bivibranchia velox* | LBP 5757 | 28123 | HQ171351 | HQ289640 | - | - | HQ289446 |
| *Hemiodus immaculatus* | LBP1725 | 12849 | HQ171246 | HQ289537 | HQ288956 | HQ289153 | HQ289344 |
|  |  |  |  |  |  |  |  |
| Hepsetidae |  |  |  |  |  |  |  |
| *Hepsetus odoe* | LBP 7527 | 35373 | HQ171387 | HQ289674 | HQ289095 | HQ289288 | HQ289480 |
|  |  |  |  |  |  |  |  |
| Lebiasinidae |  |  |  |  |  |  |  |
| *Copella nattereri* | LBP 4377 | 24148 | HQ171431 | HQ289607 | HQ289027 | HQ289220 | HQ289414 |
| *Copella nattereri* | LBP 536 | 7140 | HQ171318 | HQ289716 | HQ289139 | HQ289330 | HQ289523 |
| *Pyrrhulina australis* | LBP 3784 | 22333 | HQ171293 | - | - | HQ289197 | HQ289390 |
| *Pyrrhulina cf. zigzag* | LBP 8005 | 37473 | HQ171400 | HQ289687 | HQ289108 | HQ289301 | HQ289493 |
|  |  |  |  |  |  |  |  |
| Parodontidae |  |  |  |  |  |  |  |
| *Apareiodon affinis* | LBP 4591 | 24665 | HQ171328 | HQ289617 | HQ289037 | HQ289230 | HQ289424 |
| *Parodon nasus* | LBP 1135 | 5635 | HQ171429 | HQ289714 | HQ289137 | HQ289328 | HQ289521 |
|  |  |  |  |  |  |  |  |
| Prochilodontidae |  |  |  |  |  |  |  |
| *Prochilodus reticulatus* | LBP 6127 | 29514 | HQ171358 | HQ289647 | HQ289067 | HQ289260 | HQ289453 |
| *Semaprochilodus laticeps* | LBP 1383 | 12728 | HQ171245 | HQ289536 | HQ288955 | HQ289152 | HQ289343 |
|  |  |  |  |  |  |  |  |
| Serrasalmidae |  |  |  |  |  |  |  |
| *Catoprion mento* | LBP 7556 | 35624 | HQ171392 | HQ289679 | HQ289100 | HQ289293 | - |
| *Colossoma macropomum* | LBP 5173 | 26648 | HQ171343 | HQ289632 | HQ289052 | HQ289245 | HQ289438 |
| *Metynnis mola* | LBP 667 | 8050 | HQ171433 | HQ289718 | - | - | HQ289525 |
| *Metynnis lippincottianus* | LBP 6282 | 29688 | HQ171364 | HQ289651 | HQ289072 | HQ289265 | HQ289458 |
| *Myloplus rubripinnis* | LBP 2184 | 15570 | HQ171252 | HQ289543 | HQ288962 | HQ289159 | HQ289350 |
| *Mylossoma duriventre* | LBP 1823 | 12921 | HQ171248 | HQ289539 | HQ288958 | HQ289155 | HQ289346 |
| *Piaractus mesopotamicus* | LBP 4255 | 23803 | HQ171315 | HQ289604 | HQ289024 | HQ289217 | HQ289411 |
| *Pygocentrus cariba* | LBP 2229 | 15662 | HQ171256 | HQ289547 | HQ288966 | HQ289163 | HQ289354 |
| *Serrasalmus maculatus* | LBP 3698 | 21836 | HQ171285 | - | HQ288995 | HQ289189 | HQ289382 |
| *Serrasalmus spilopleura* | LBP 3499 | 20169 | HQ171281 | HQ289570 | HQ288991 | HQ289185 | HQ289378 |
| *Tometes trilobatus* | LBP 9072 | 42585 | HQ171425 | HQ289711 | HQ289133 | - | HQ289518 |
|  |  |  |  |  |  |  |  |
| Cypriniformes |  |  |  |  |  |  |  |
| *Carassius auratus* | LBP 9215 | 43160 | HQ171427 | HQ289713 | HQ289135 | - | - |
| *Gyrinocheilus sp*.^1^ | AMNH 233433 |  | AY788015 | AY791399 | - | - | AY804074 |

1- Calcagnotto *et al.* [32]; 2- Javonillo *et al.* [33].
